# Peer-friendship Networks and Self-harm among Adolescents from Inner-city Schools: A Social Network Study

**DOI:** 10.1007/s10964-025-02264-y

**Published:** 2025-10-03

**Authors:** Holly Crudgington, Rachel Blakey, Molly Copeland, Charlotte Gayer-Anderson, Samantha Davis, Katie Lowis, Esther Putzgruber, Thai-sha Richards, Jonas Kitisu, Adna Hashi, Karima Shyan Clement-Gbede, Niiokani Tettey, Daniel Stanyon, Alice Turner, Lynsey Dorn, Seeromanie Harding, Kamaldeep Bhui, Vanessa Pinfold, Gemma Knowles, Craig Morgan

**Affiliations:** 1 ESRC Centre for Society and Mental Health, King’s College London, London, UK; 2 Health Service and Population Research Department, Institute of Psychiatry, Psychology and Neuroscience, King’s College London, London, UK; 3 Department of Experimental Psychology, University of Oxford, Oxford, UK; 4 Population Health Sciences and MRC Integrative Epidemiology Unit, University of Bristol, Bristol, UK; 5 Department of Sociology, University of Notre Dame, Notre Dame, IN USA; 6 Department of Population Health Sciences, School of Life Course and Population Sciences, Faculty of Life Sciences & Medicine, King’s College London, London, UK; 7 Department of Psychiatry, University of Oxford, Oxford, UK; 8 McPin Foundation, London, UK

**Keywords:** Adolescence, Self-harm, Social networks, Peers

## Abstract

Peer-relationships are critically important for adolescent behavior, but how peer-friendship network composition and structure influence adolescent self-harm is less clear. The aim of this cross-sectional study was to explore the association between in-school peer-friendship networks, gender, and self-harm among inner-city adolescents. Participants were 2203 adolescents (mean age = 12.5 years, SD = 1.0; 53% girls) attending inner-city south London schools. Each adolescent nominated friends within their school year to construct sociometric peer-friendship networks and reported on lifetime self-harm. Mixed-effects logistic regression was used to estimate the effects of a comprehensive array of peer-network metrics on self-harm in the sample overall and by gender. Having friends who report self-harm, network over-integration (bridging, popularity), and social isolation (network under-integration) increased odds of self-harm, while sociality and high friendship group density reduced odds. Odds ratios did not vary by gender. The findings indicate that peer-network composition, particularly if friends self-harm, and over- and under-integration in wider peer-networks, may influence early adolescent self-harm, among both boys and girls.

## Introduction

Self-harm, defined as any intentional self-poisoning or self-injury regardless of suicidal intent, is a widespread health challenge in adolescence (National Institute for Health and Care Excellence, 2022). Theoretical models of self-harm suggest it can serve social functions (e.g., communicating distress) (Nock & Prinstein, 2004), which may be particularly relevant in adolescence, a developmental period marked by a “social shift” towards peers (Brown & Larson, 2009). While much research suggests peer-relationships matter for self-harm, most studies focus on adolescents’ perceptions of their peers, which can be biased (Quigley et al., 2017a). Sociometric network studies, where adolescents nominate in-school friends and both they and their peers self-report behaviors, offer a more objective approach by capturing peer-reported behaviors alongside social network structure. This approach is valuable because schools are key social contexts where both peer behaviors (network composition) and the patterns of connections among peers (network structure) have been linked to health and mental health outcomes (Eccles & Roeser, 2011). However, despite the potential to advance understanding of the social aspects of self-harm, studies that combine sociometric and self-harm data remain scarce. In addition, evidence suggests self-harm and social relationships vary geographically (Polling et al., 2019), and highly urbanized environments, such as inner-city London, may have distinct stressors and peer dynamics that shape how peer-networks relate to self-harm. Therefore, localized investigation in such settings is needed. This study addresses these gaps by exploring how the composition and structure of sociometric in-school peer-friendship networks and gender relate to self-harm among a diverse sample of inner-city London youth.

### Adolescent Self-harm and Peers

Self-harm is a complex and multi-faceted behavior that typically begins in early adolescence, around 12–13 years of age (Gillies et al., 2018). Around 17% of young people report having self-harmed at least once in their lifetime, and the behavior appears to be increasing, particularly among adolescent girls (Morgan et al., 2017). Theoretical models of self-harm, like the four-function model of non-suicidal self-injury (NSSI), suggest it can serve both internal (e.g., emotional regulation) and social functions (e.g., interpersonal). For example, to produce desired social situations, or to avoid aversive social events and interpersonal pressure (Nock & Prinstein, 2004). Social aspects of self-harm are important to consider in adolescence, as this is a period characterized by rapid social development and the increasing importance of peers (Brown & Larson, 2009).

One way that peer-relationships may relate to self-harm is via the behaviors and experiences of peers. Research shows that adolescents who know of self-harm among their peers are more likely to report similar behaviors themselves (Jarvi et al., 2013). This association may be explained by several processes, such as social learning and modeling, whereby adolescents observe or internalize self-harm as a coping strategy through their peers (Nock, 2009). Broader research on peers also shows that self-harm can be openly discussed among friends and used as a means of social bonding (Bilello, Townsend, Broome, & Burnett Heyes, 2024).

### Sociometric Network Studies

However, much of the existing research on friends’ self-harm tends to rely on broad terms like “friend” without fully defining the relationship (e.g., peer-friendship) or context (e.g., school), and adolescents’ perception of friends’ behavior which can be prone to bias (Quigley et al., 2017b). An approach that addresses these limitations is to collect data on self-harm and link this with the collection of sociometric data (i.e., data on the relationships between individuals in a defined boundary) via a friendship survey which asks who the adolescent currently views as a friend in school. Combining this data together allows for the study of social structures among peers as well as adolescents’ own subjective reports of self-harm. This is important, as peers’ reports of behaviors and peer social structures have been linked to adolescent health and mental health outcomes (Crudgington et al., 2023; Kawachi & Berkman, 2001).

### Peer-network Composition

A recent systematic review identified a small but growing number of sociometric studies focused on self-injurious thoughts and behaviors and peer-friendship networks in adolescence (Crudgington et al., 2023). There was evidence that friends’ reports of self-cutting (a specific form of self-harm) and non-suicidal self-injury (NSSI) were positively associated with adolescents’ own similar behavior. These findings underscore the importance of sociometric peer-network composition, as measured by friends’ own self-report of behavior, as a potential source of risk for adolescent self-harm. However, it is still unclear whether peers self-harm relates to self-harm as defined in the current study (i.e., self-harm irrespective of suicidal intent), in diverse inner-city samples, or independent from peer-network structure.

### Peer-network Structure (Integration)

Beyond who adolescents are friends with, sociometric research has shown that the broader structure of peer-friendship networks also plays an important role in shaping mental health (Cotterell, 2013). That is, adolescents’ degree of integration within peer networks - including both over- and under-integration - may have protective or detrimental effects on wellbeing (Crudgington et al., 2023).

One important aspect of peer-network integration is “sociality” (i.e., out-degree, a measure of the number of peers an adolescent nominates as friends). Higher sociality is associated with reduced self-cutting behavior (Copeland et al., 2019) and suicide attempts and ideation (Wyman et al., 2019) among samples of adolescents in the United States. Sociality might reflect pro-social orientation, and signal a sense of belonging and identity among peers which may be protective for wellbeing. However, whether sociality relates to self-harm more broadly (beyond self-cutting and irrespective of suicidal intent) remains unclear.

Integration within peer-friendship groups is also important to consider. One measure of this is “friendship group density” which reflects how inter-connected an adolescent’s nominated friends are with each other. Although being part of a densely connected friendship group may reflect availability of social support and be beneficial for wellbeing, it may also have nuanced, non-linear effects, on mental health. For example, one study found that having a very large or very small friendship group was positively associated with depressive symptoms among adolescents (Falci & McNeely, 2009). However, for girls, the negative effects of having many friends only appeared when those friends were poorly connected to each other (low density). By contrast, for boys, having many friends who were well connected to each other (high density) was linked to increased depressive symptoms. Low density (i.e., sparsely connected) friendship groups may reflect reduced collective support or a lack of cohesion among friends, whereas high density friendship groups, while potentially supportive, may create relational strain or interpersonal stress (Falci & McNeely, 2009). Although friendship group density has yet to be examined in the context of adolescent self-harm, it is possible that comparable mechanisms may underlie associations between high and low friendship group density and self-harm among youth.

### Network Over-integration

In addition, some sociometric network measures that capture network *over*-integration - structural positions that reflect being highly embedded in the wider peer-network – have been found to have adverse effects on mental health. For example, “bridging” (i.e., betweenness centrality, a measure of how often an adolescent is positioned between two others in a network as a “bridge” connecting them) is associated with increased self-cutting behavior among adolescents in the US (Copeland et al., 2019) and with increased internalizing distress, particularly among adolescent girls (Carboni & Gilman, 2012). Although high betweenness may reflect access to social support and influence through brokerage - which could be beneficial to wellbeing – these studies suggest it may also come with challenges. Acting as a “bridge” between friendship groups may create social pressure, contributing to emotional distress, which may indirectly impact self-harm (Copeland et al., 2019). Similarly, while popularity (i.e., the number of incoming nominations received by an adolescent) reflects high status among peers and may be socially desirable, this metric has been found to be associated with negative behavioral outcomes among youth such as depressive symptoms (Reynolds & Crea, 2015). Popular adolescents may have to navigate complex peer dynamics and experience pressure to maintain their social status. Thus, consistent with prior work that considers high bridging and popularity as forms of network over-integration that can be harmful to health, these network positions may create social strain and stress, which may be taxing on mental health, and indirectly relate to self-harm.

### Network Under-integration

In addition to risks of *over-*integration in networks, *under*-integration (i.e., a lack of connection) also has associated risks. Indeed, a wealth of research and theory highlight the association between perceived social isolation and poor mental health (Christiansen et al., 2021). Perceived social isolation can lead to feelings of rejection, loneliness, and emotional distress, which are known risk factors for depression, anxiety, and self-harm (Liu et al., 2020). *Sociometric* social isolation (i.e., adolescents who have no or very few ties) reflects adolescents’ actual disconnection from their peer-network regardless of how they feel. Of the few studies that have explored sociometric isolation in relation to adolescent mental health, it has been found to be associated with increased suicide ideation and attempts particularly among adolescent girls (Crudgington et al., 2023). Being an “isolate” within peer-friendship networks might lead to feelings of low self-worth, and in turn, suicidal ideation (Bearman & Moody, 2004). It is plausible that social isolation may be associated with adolescent self-harm; however, this remains un-explored.

### Gender and Context

Gender is important to consider in research on self-harm and peer-networks, as self-harm is more common among adolescent girls compared with boys, and gender homophily (i.e., the tendency to be linked to those who share similar characteristics) is a common feature of adolescent peer-friendship networks (Goodreau et al., 2009). Evidence also indicates that network integration may relate differently to mental health by gender (Crudgington et al., 2023; Falci & McNeely, 2009). This aligns with a developmental trade-off model, which suggests that friendships may have different emotional costs for girls compared with boys (Rose & Rudolph, 2006). Girls may be more likely to engage in pro-social interactions with friends characterized by self-disclosure compared with boys who, overall, may receive fewer emotional provisions from friendships and may interact in larger groups. These gendered patterns in social relationships suggest that the structure and composition of peer-friendship networks may relate to self-harm differently for boys and girls. However, few studies have formally tested gender moderation of peer-network metrics on mental health or self-harm, so this remains unclear.

In addition, some evidence suggests that self-harm varies by other social factors, like ethnic group (Farooq et al., 2021), and place (e.g., variation in self-harm rates within urban areas) (Polling et al., 2019) as do social relationships (Fischer, 1982). Thus, it is important to consider how peer-networks may relate to self-harm in diverse social contexts, such as inner-city London schools, where self-harm is prevalent (~15%) (Knowles et al., 2021) but social environments, stressors, and peer dynamics might alter how peer-networks relate to self-harm. Indeed, prior research on inner-city London youth found that ethnic group differences in conduct problems were partly explained by differential exposure to troublesome friends, highlighting how the composition of peer networks may shape behavioral outcomes in this context (Blakey et al., 2021). In addition, other work finds that peer-network structure relates to mental health differently across racial and ethnic groups (Copeland & Kamis, 2022). While not the primary focus of this study, extending network research to the context of inner-city London schools helps to explore whether established associations for peer-networks, mental health, and self-harm hold across different social contexts.

## The Current Study

Despite evidence suggesting peer-relationships matter for self-harm, and that peer-networks matter for mental health more broadly, self-harm has rarely been studied through the lens of sociometric peer-networks, which are important to consider given the theorized social functions of this behavior in adolescence. This study addresses a critical gap in the literature by empirically testing how multiple dimensions of sociometric in-school peer-friendship networks (both composition and structure) relate to adolescent self-harm, and whether there are gender differences. Moreover, this study uses data from a large, diverse, cohort of youth from inner-city London which can offer insights into self-harm in a population that remains under-represented in existing network research. Based on prior theory and literature, five hypotheses were tested. First, *friends self-harm* (network composition) was hypothesized to be associated with increased odds of adolescents own self-harm (Hypothesis 1). Second, consistent with prior work that suggests having friends to name may be protective for wellbeing including self-cutting, *sociality* (out-degree) was hypothesized to be associated with reduced odds of self-harm (Hypothesis 2). Third, in line with the adolescent mental health literature, which suggests highly interconnected friendship groups and network *over*-integration is associated with mental distress, *high friendship-group density* (vs. medium), *popularity* (in-degree), and *bridging* (betweenness centrality) were hypothesized to be associated with increased odds of self-harm (Hypothesis 3). Fourth, consistent with prior research that suggests low density friendship groups and network *under*-integration may be linked to increased mental distress and suicidal behavior, *low friendship-group density* (vs. medium) and *isolation* (i.e., having 0 or 1 friend) were hypothesized to be associated with increased odds of self-harm (Hypothesis 4). Fifth, peer-friendships may serve different functions for girls and boys, thus gender was hypothesized to moderate any observed associations, such that associations between network metrics and self-harm would be in the same direction for both genders but stronger among girls compared with boys (Hypothesis 5).

## Methods

### Participants and Procedure

The Resilience Ethnicity and Adolescent Mental Health (REACH) study is an accelerated cohort study of adolescents from 12 mainstream secondary schools in two ethnically and socioeconomically diverse boroughs of inner-city London, UK (Knowles et al., 2022). Briefly, schools included in REACH were selected to be representative of the 38 mainstream secondary schools within Southwark and Lambeth based on a) the number of pupils in receipt of free-school meals (a marker of socioeconomic status) and b) the number of pupils from minority ethnic groups. These two boroughs were selected as they are highly socioeconomically and ethnically diverse. For example, both boroughs are among the top 20% most deprived boroughs in the UK, with prevalence of mental health problems among adults in these boroughs twice that of national prevalence estimates (Hatch et al., 2012; Lambeth Council, 2016). The full cohort profile and a paper on prevalence of mental distress and self-harm among the cohort have been published elsewhere (Knowles et al., 2021, 2022). Ethical approval for the study was granted from the Psychiatry, Nursing and Midwifery Research Ethics Subcommittee (PNM-RESC), King’s College London (ref:15/162320).

At Time 1 (T1), between February 2017 and January 2018, 4945 students were invited to take part and 4353 (88%) gave informed consent and completed an electronic questionnaire including items on their mental health and social networks. Students completed the e-questionnaire in a classroom setting (~1hr), with trained researchers present. Three school-based cohorts were constructed at T1: Cohort 1 (C1), school year 7 (ages 11–12); Cohort 2 (C2), school year 8 (ages 12–13 years); and Cohort 3 (C3), school year 9 (ages 13–14). Although REACH has three waves of data, this study is cross-sectional due to limitations in consistent measures across waves and the availability of only T1 peer-friendship networks, providing a first step in examining newly constructed sociometric data.

### Sample Selection

Three schools were excluded from analyses because two were (originally) pilot schools where self-harm questions were not administered at baseline, and one did not collect sufficient social network data. Therefore, a sub-sample of 3378 adolescents from nine schools at T1 were eligible to be included in this study. Of those eligible, 2969 participated (87.9%) and the most common reason for missing data was pupil absence (n, 241). Information on network missingness is available in Supplementary Table S2. Participants with missing data on variables of interest were listwise deleted culminating in a complete case analysis sample of 2203 participants. A comparison of this analytical sample relative to the eligible (n, 3378) and full REACH baseline sample (n, 4353) is available in Supplementary S5. Briefly, compared with the REACH baseline sample, the analytic sample had a slight under-representation of students on free-school meals (19% vs. 24%) and Black Caribbean (13% vs. 16%) adolescents, and a slight over representation of girls (53% vs. 51%), Black African (29% vs. 26%) and White British (17% vs. 14%) adolescents.

### Peer-friendship Networks

**Fig. 1:**
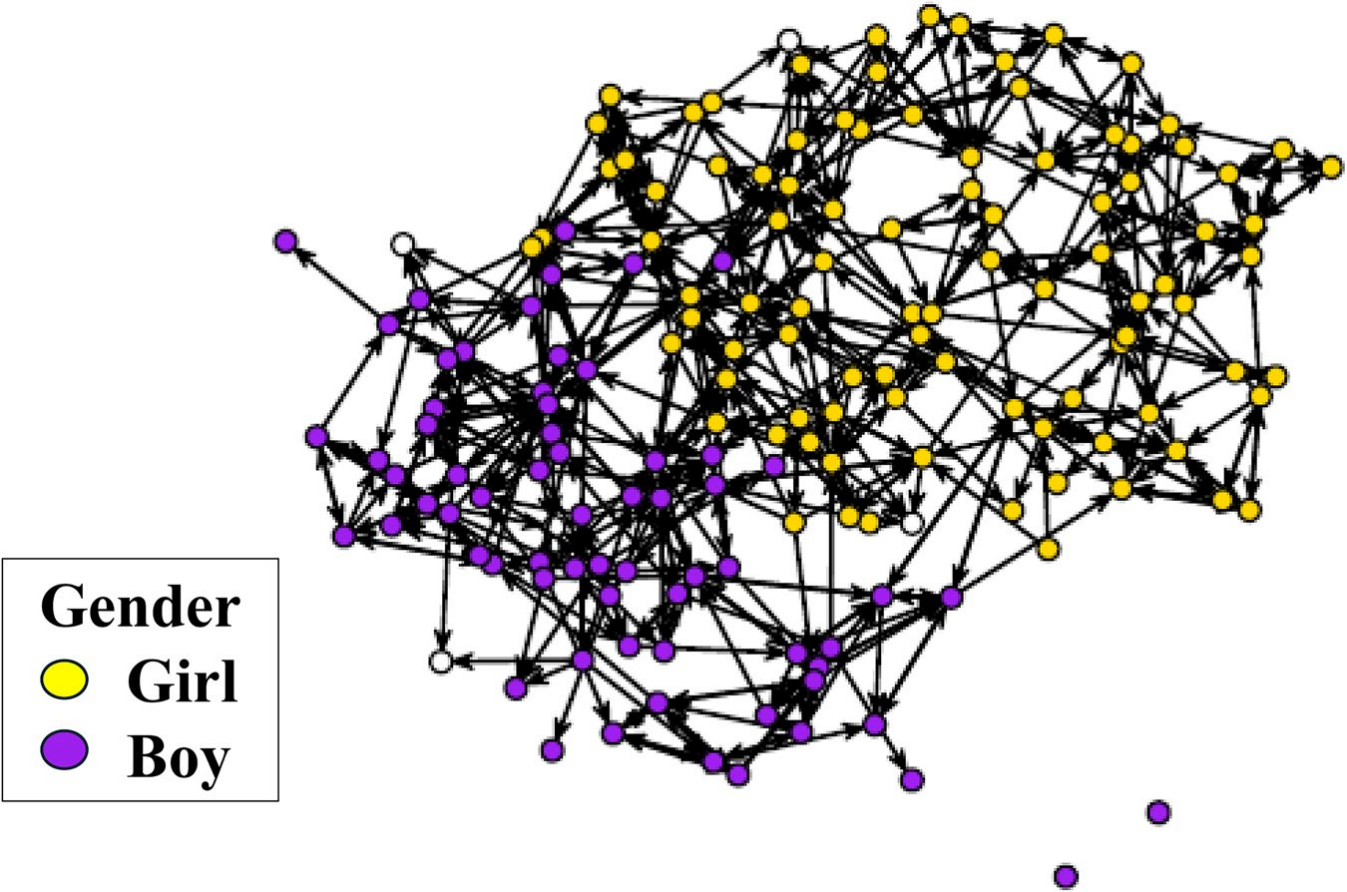
Sociogram of a year 7 friendship network in REACH visualized with the Fruchterman-Reingold algorithm in R. The circles represent adolescents in the network and the arrows represent directed friendship nominations between them. White circles represent adolescents with missing data on gender

Adolescents nominated up to five friends within their school year who they spent the most amount of time with in the last six months. Friendship nominations were matched against the official first and last names of the students in the REACH database to construct sociometric friendship networks of school year groups (93.9% match rate). For this study based on nine schools, adolescents were part of 25 separate networks: eight schools with three year groups (years 7, 8, and 9) [8*3 = 24 networks], one (newly created at the time) school with one year 7 group [1 network]. Each network was described (Table S2) and visualized as a sociogram (see Fig. 1 and Figures in S4). Social Network Analysis was used to calculate seven peer-network metrics that reflect network composition (friends’ self-harm) and network integration. Formal network terminology is used below: each adolescent is referred to as the “ego”, and their immediate send and receive nominations constitute their “ego-network” from within the broader sociometric network.

### Network Measures

#### Friends’ self-harm

A categorical variable that reflects the number of friends in an adolescent’s friendship group (send and receive ego-network) that reported yes to lifetime self-harm.

#### Sociality

Measured with “out-degree” i.e., the number of outgoing nominations sent by an adolescent.

#### Popularity

Measured with “in-degree” i.e., the number of incoming nominations received by an adolescent.

#### Bridging

Measured with “betweenness centrality”, a measure that reflects the number of times an adolescent (ego) falls on the shortest path (geodesic) between other adolescents in the network and higher scores reflect adolescents that bridge otherwise disconnected friendship groups.

#### Friendship-group density

Measured with send and receive ego-network density, a measure of how interconnected an adolescent’s friendship group is, calculated as a ratio of the number of ties present in the ego-network divided by the total number of possible ties, including ego in the calculation. Ego-network density ranges from 0 – 1, and this was categorized this into thresholds of low (0 – 0.3), medium (0.31 – 0.59), and high (0.6 – 1). Further information about calculation of sociometric variables is available in Supplementary S3.

#### Isolation

A binary variable where “1” reflects adolescents with 0 or 1 connection to the peer-network (regardless of direction) and “0” reflects all other adolescents.

#### Friendship-group size

Measured with send and receive ego-network size, i.e., an adolescent’s total number of connected friends. Each friendship is counted only once, whether it is sent only, received only, or reciprocal.

### Other Measures

#### Self-harm

Lifetime self-harm was measured using one self-report item from the Development and Adolescent Wellbeing Assessment (DAWBA): “Over the whole of your lifetime, have you ever tried to harm or hurt yourself?” dichotomized into 1= yes, 0 = no (Goodman et al., 2000).

#### Age and cohort

Age was measured in years, and cohort is a categorical variable that reflects the year group adolescents were in: Cohort 1 (year 7), Cohort 2 (year 8), and Cohort 3 (year 9).

#### Free school meal status

Is a binary measure used as a proxy for socioeconomic status, where “1” reflects adolescents who self-report they are in receipt of free-school meals and “0” reflects all other adolescents. Eligibility for free-school meals in London (and throughout the UK) is based on the financial situation of the pupil’s family. Pupils are eligible to receive free-school meals if their parents/carers are in receipt of one or more means-tested government benefits (before April 1st, 2018) and if their family’s annual income was no more than £16,190 (after April 1st, 2018) (Department for Education, UK Government, 2023).

#### Ethnic group

Measured as pupils self-report of their ethnic group based on categories from the 2011 UK census (Office for National Statistics, 2012). This consisted of Black African, Black Caribbean, White British, Indian Pakistani, Bangladeshi, Other Black, Mixed/Multiple, Any Other, Non-British White, Mixed White and Black, Latin American (with White as the reference group).

### Statistical Analyses

Networks were constructed, visualized, and sociometric variables calculated in R (v4.2.1), using the statnet (v2019.6) and igraph (v1.3.5) packages. All analyses were performed in STATA 17.0. Given that adolescents are clustered by school and students within the same school are likely to be similar to each other, mixed-effects multi-level logistic regression, with a random effect for school to adjust for clustering within school, was used to estimate odds ratios for associations between sociometric variables and lifetime self-harm.

Three sets of models were estimated: (a) unadjusted [Model 1], (b) adjusted for a priori potential confounders: gender, free-school meal status, ethnic group, and age [Model 2], and (c) further adjusted for an additional network control variable where applicable [Model 3]. That is: (i) for models in which friends’ self-harm, bridging, or friendship group density were the exposure of interest, friendship group size was controlled for; and (ii) for models in which popularity and sociality were the exposures of interest, these two metrics were added to the model simultaneously (i.e., in-degree adjusted for out-degree, and vice versa).

To explore gender differences, first gender-stratified models were estimated to explore patterns of associations separately for boys and girls. Second, effect modification by gender was formally tested by adding an interaction term for gender*social network variables in unadjusted and adjusted models in the whole sample. Post-estimation linear combinations of model coefficients were used to estimate effects for boys and girls separately, directly from the interaction models (Table S7). In the main paper tables, estimates are presented stratified by gender.

### Approach to Interpretation

In line with recent discussion and recommendations that recognize the limitations of interpretations primarily based on statistical significance (Wasserstein et al., 2019), the interpretation of results is focused on the magnitude of the effect size (Odds Ratios [ORs]) and precision of effects (95% Confidence Intervals [CIs]).

### Youth Involvement

Alongside a dedicated youth involvement program that has shaped REACH from its inception (Richards & Robotham, 2022), five adolescents work as co-researchers on the project, the Young Person’s Community Champions (YPCC). The YPCC bring their unique skills and perspectives to the team. For this study, the YPCC were invited to participate in a study meeting after analyses to share their insights on this study’s findings. This was a short (~45 min) session where they were presented the results and asked to feedback, particularly on anything they found striking or interesting. Some of their feedback on the results (with permission from the YPCC) has been integrated into the main discussion (and in Supplementary S10).

## Results

### Participant and Network Characteristics

Tables 1 and 2 show participant sociodemographic and network characteristics overall and by gender. The sample (n, 2203) consisted of 53% girls, aged between 11–14 years (mean age 12.5, SD: 1.0) with 19% having received free school meals. The ethnic group breakdown was 29% Black African, 17% White British, 13% Black Caribbean with < 10% for all other ethnic groups. Around 14% reported yes to lifetime to self-harm, and this was more prevalent among girls (16%) compared with boys (11%).

**Table 1:** Participant Characteristics

	Total	Boys	Girls	*p*
	(n, 2203)	(n, 1026)	(n, 1177)	
Gender n(%)				
Boy	1026 (46.6)	—	—	
Girl	1177 (53.4)	—	—	
Age (M±S.D)	12.5 (1.0)	12.6 (1.0)	12.4 (1.0)	<0.001
Cohort n(%)				0.037
Cohort 1 (Year 7)	733 (33.3)	341 (33.2)	392 (33.3)	
Cohort 2 (Year 8)	720 (32.7)	311 (30.3)	409 (34.8)	
Cohort 3 (Year 9)	750 (34.0)	374 (36.5)	376 (32.0)	
Ethnic group n(%)				0.059
Any Other	145 (6.6)	76 (7.4)	69 (5.9)	
Black African	639 (29.0)	293 (28.6)	346 (29.4)	
Black Caribbean	291 (13.2)	120 (11.7)	171 (14.5)	
Indian, Pakistani, Bangladeshi	112 (5.1)	44 (4.3)	68 (5.8)	
Latin American	85 (3.9)	44 (4.3)	41 (3.5)	
Mixed White & Black	188 (8.5)	93 (9.06)	95 (8.07)	
Mixed/Multiple	129 (5.9)	57 (5.6)	72 (6.1)	
Non-British White	174 (7.9)	86 (8.4)	88 (7.5)	
Other Black	68 (3.1)	24 (2.3)	44 (3.7)	
White British	372 (16.9)	189 (18.4)	183 (15.5)	
Receiving free school meals n(%)				
No	1775 (80.6)	839 (81.8)	936 (79.5)	0.183
Yes	428 (19.4)	187 (18.2)	241 (20.5)	
Lifetime self-harm n(%)				<0.001
No	1902 (86.3)	915 (89.2)	987 (83.9)	
Yes	301 (13.7)	111 (10.8)	190 (16.1)	

χ2 analyses were used for comparison of categorical variables, the t-test was used for comparison of continuous variables. Comparing boys with girls

*M* mean, *S.D* standard deviation, *n* number, *p* p value

**Table 2:** Participant Network Characteristics

	Total (n, 2203)	Boys (n, 1026)	Girls (n, 1177)	*p*	Min – Max
Friends’ self-harm, n(%)				<0.001	0–3
0 friends report self-harm	1215 (55.2)	612 (59.6)	603 (51.2)		
1 friend reports self-harm	674 (30.6)	297 (29.0)	377 (32.0)		
2 friends report self-harm	230 (10.4)	88 (8.6)	142 (12.1)		
≥3 friends report self-harm	84 (3.8)	29 (2.8)	55 (4.7)		
Isolated, n(%)				<0.001	0–1
No	2097 (95.2)	958 (93.4)	1139 (96.8)		
Yes	106 (4.8)	68 (6.6)	38 (3.2)		
Popularity (M±S.D)	3.4 (2.2)	3.1 (2.3)	3.5 (2.1)	<0.001	0–14
Sociality (M±S.D)	4.0 (1.7)	3.9 (1.8)	4.2 (1.5)	0.001	0–5
Bridging (M±S.D)^a^	4.2 (6.2)	4.1 (6.1)	4.3 (6.3)	0.553	0–59
Friendship group density n(%)				<0.001	0–3
Low density (0–0.3)	798 (36.2)	486 (47.4)	312 (26.5)		
Medium density (0.31–0.59)	1189 (54.0)	489 (47.7)	700 (59.5)		
High density (0.6–1)	216 (9.8)	51 (5.0)	165 (14.0)		
Friendship group size (M+SD)	5.6 (2.1)	5.6 (2.3)	5.5 (2.0)	0.568	0–15

χ2 analyses were used for comparison of categorical variables, the t-test was used for comparison of continuous variables. Comparing boys with girls

*M* mean, *S.D* standard deviation, *n* number, *Min* max values are from total sample, *p* p value

^a^Raw bridging (betweenness) values ranged from 0 to 5880. To aid interpretation and analysis, bridging values were divided by 100, with a range of 0 to 59 and this scaled version was used in the main analyses

Nearly half (45%) of adolescents were friends with at least one person who reported self-harm, and the mean friendship group size was six. Around 5% of adolescents were isolated from the peer-network. Correlations between network variables are available in Table S1 and descriptive statistics of network characteristics by cohort and ethnic group are provided in Table S6 for completeness and information.

Across all model specifications, being a girl (vs. boy) was consistently associated with higher odds of lifetime self-harm (ORs ranged from 1.21–1.25 across models). In addition, age (ORs 1.21–1.25) and receiving free-school meals (ORs 1.39–1.57) were associated with increased odds of self-harm (ORs for all covariates across all model specifications are available in Supplementary S9).

### Peer-network Composition and Self-harm

#### Friends’ self-harm

Table 3 provides strong evidence that friends’ self-harm was associated with increased odds of lifetime self-harm (supporting Hypothesis 1). For example, for adolescents that had at least one friend who reported self-harm, odds of lifetime self-harm increased by 63% (aOR 1.63, 95%CI 1.25, 2.13) compared with adolescents that had no friends report self-harm. Additionally, the greater the number of friends who reported self-harm, the greater the estimated odds of self-harm. For adolescents that had only one friend report self-harm, odds of self-harm increased by 32% (aOR 1.32 95%CI 0.98, 1.78). However, for adolescents who had two friends report self-harm, there was more than a two-fold increase in odds of self-harm (aOR 2.35, 95%CI 1.60, 3.46) and having three or more friends report self-harm was associated with an almost four-fold increase in odds of self-harm (aOR 3.72, 95%CI 2.20, 6.28).

**Table 3:** Estimated Odds Ratios for the Effects of Sociometric Network Variables on Lifetime Self-harm among Adolescents Overall

	Model 1		Model 2		Model 3*^ab^	
	uOR (95%CI)	*p*	aOR (95%CI)	*p*	aOR (95%CI)	*p*
Friends’ self-harm^*a^						
0 friends report self-harm	*Ref*		*Ref*		*Ref*	
At least 1 friend reports self-harm	1.72 (1.34, 2.21)	0.000	1.63 (1.26, 2.10)	0.000	1.63 (1.25, 2.13)	<0.001
Friends’ self-harm^*a^						
0 friends report self-harm	*Ref*		*Ref*		*Ref*	
1 friend reports self-harm	1.37 (1.03, 1.82)	0.029	1.29 (0.96, 1.72)	0.083	1.32 (0.98, 1.78)	0.064
2 friends report self-harm	2.30 (1.58, 3.32)	<0.001	2.24 (1.55, 3.24)	0.000	2.35 (1.60, 3.46)	<0.001
≥3 friends report self-harm	3.86 (2.31, 6.44)	<0.001	3.53 (2.13, 5.85)	0.000	3.72 (2.20, 6.28)	<0.001
Friendship-group density^*a^						
Medium density	*Ref*		*Ref*		*Ref*	
Low density	0.86 (0.66, 1.12)	0.281	0.94 (0.72, 1.24)	0.710	0.93 (0.70, 1.23)	0.639
High density	0.59 (0.36, 0.97)	0.039	0.50 (0.30, 0.83)	0.007	0.51 (0.31, 0.85)	0.010
Bridging^*a^	1.02 (1.01, 1.04)	0.008	1.02 (1.01, 1.04)	0.007	1.02 (1.00, 1.05)	0.014
Popularity^*b^	1.04 (0.99, 1.10)	0.082	1.04 (0.98, 1.11)	0.088	1.05 (0.99, 1.12)	0.051
Sociality^*b^	0.97 (0.90, 1.04)	0.445	0.96 (0.89, 1.04)	0.376	0.95 (0.88, 1.02)	0.192
Isolated						
No	*Ref*		*Ref*			
Yes	1.73 (1.05, 2.83)	0.029	1.86 (1.13,3.08)	0.016	—	—
	n, 2203		n, 2203		n, 2203	

All models adjusted for clustering at the school level. Model 1 is an unadjusted bivariable model of each network metric with self-harm. Model 2 is an adjusted model of each network metric and self-harm adjusted for gender, age, free school meal status, ethnic group. Model 3 is an adjusted model of each network metric and self-harm adjusted for gender, age, free school meal status, ethnic group, and a network control variable (*a [adjusted for friendship group size], *b [popularity adjusted for sociality, sociality adjusted for popularity]

*uOR* unadjusted odds ratio, *aOR* adjusted odds ratios

### Peer-network integration and Self-harm

#### Having friends to nominate

There was partial support for Hypothesis 2 (Table 3). There was, at most, a small association between sociality and lower odds of self-harm (aOR 0.95, 95% CI 0.88, 1.02).

#### High friendship group density and network over-integration

There was partial support for Hypothesis 3 (Table 3). The strongest evidence was for high-friendship group density. Contrary to expectations, being part of a high-density friendship group (vs medium) was associated with 49% lower odds of lifetime self-harm (aOR 0.51, 95%CI: 0.31, 0.85). However, in line with expectations, there was a small association between popularity (aOR 1.05, 95%CI 0.99, 1.12) and bridging (aOR 1.02, 95%CI 1.00, 1.05) and increased odds of self-harm. For bridging, notably, the predicted probabilities of self-harm were higher for those who scored highly on this metric. For example, holding all demographic variables and friendship group size constant at the mean (consistent with model 3), the predicted probability of self-harm for adolescents with a bridging value of 0 was 0.12 (95%CI 0.10, 0.14), the predicted probability for adolescents who scored the mean bridging value was 0.13 (95%CI 0.11, 0.15), whereas the predicted probability for those who scored at the 99th percentile (i.e., score of 29.5) was 0.21 (95%CI 0.13, 0.30).

#### Low friendship group density and network under-integration

There was partial support for Hypotheses 4 (Table 3). The strongest evidence was for isolation i.e., being isolated (vs not) was associated with an almost two-fold increase in odds of self-harm (aOR 1.86, 95% CI: 1.13, 3.08). However, contrary to expectations, being part of a low-density friendship group (vs medium) was not associated with self-harm – or with, at most, a small, reduced odds of self-harm (aOR 0.93, 95% CI: 0.70, 1.23).

### Gender

In gender stratified models (Tables 4, 5) estimated effects were largely similar for girls and boys, with only a few suggestions of potential gender differences. For example, for friends’ self-harm, among girls, having one friend report self-harm was associated with a modest 47% increase in odds of self-harm (aOR 1.47, 95%CI 1.00, 2.15), but a small or no increase among boys (aOR 1.05, 95%CI 0.64, 1.72; p for interaction 0.239). However, for 2 friends, and 3+ or more friends reporting self-harm, the patterns between boys and girls were similar (i.e., a modest to large increases in odds of self-harm). For network under-integration, among boys, being isolated (vs. not) was associated with an almost 3-fold increase in odds of self-harm (aOR 2.69, 95%CI 1.41, 5.11) but a small or no increase among girls (aOR 1.04, 95%CI 0.44, 2.46, *p* value for interaction: 0.134). However, all interaction term *p* values were >.05 (Table S7). Thus, contrary to Hypothesis 5, gender did not formally moderate any observed observations i.e., data are consistent with the effect of peer-friendship network metrics on self-harm being similar for boys and girls.

**Table 4:** Estimated Odds Ratios for the Effects of Sociometric Network Variables on Lifetime Self-harm among Girls Only

	Model 1		Model 2		Model 3*^ab^	
	uOR (95%CI)	*p*	aOR (95%CI)	*p*	aOR (95%CI)	*p*
Friends’ self-harm^*a^						
0 friends report self-harm	*Ref*		*Ref*		*Ref*	
At least 1 friend reports self-harm	1.87 (1.35, 2.59)	<0.001	1.84 (1.32, 2.57)	<0.001	1.76 (1.24, 2.50)	0.001
Friends’ self-harm^*a^						
0 friends report self-harm	*Ref*		*Ref*		*Ref*	
1 friend reports self-harm	1.54 (1.07, 2.22)	0.020	1.49 (1.02, 2.16)	0.035	1.47 (1.00, 2.15)	0.044
2 friends report self-harm	2.34 (1.46, 3.74)	<0.001	2.49 (1.56, 3.96)	<0.001	2.43 (1.49, 3.97)	<0.001
≥3 friends report self-harm	3.67 (1.94, 6.94)	<0.001	3.58 (1.92, 6.69)	<0.001	3.49 (1.82, 6.68)	<0.001
Friendship-group density^*a^						
Medium density	*Ref*		*Ref*		*Ref*	
Low density	0.93 (0.64, 1.33)	0.693	1.01 (0.70, 1.46)	0.928	0.97 (0.67, 1.41)	0.900
High density	0.54 (0.31, 0.95)	0.033	0.51 (0.29, 0.91)	0.024	0.55 (0.31, 0.99)	0.047
Bridging^*a^	1.01 (0.99, 1.04)	0.127	1.01 (0.99, 1.04)	0.122	1.01 (0.98, 1.03)	0.384
Popularity^*b^	1.06 (0.99, 1.14)	0.082	1.07 (0.99, 1.15)	0.057	1.08 (1.00, 1.17)	0.041
Sociality^*b^	0.97 (0.88, 1.08)	0.674	0.98 (0.89, 1.09)	0.780	0.95 (0.86, 1.06)	0.424
Isolated						
No	*Ref*		*Ref*			
Yes	1.06 (0.45, 2.50)	0.877	1.04 (0.44, 2.46)	0.090	—	—
	n, 1177		n, 1177		n, 1177	

All models adjusted for clustering at the school level. Model 1 is an unadjusted bivariable model of each network metric with self-harm. Model 2 is an adjusted model of each network metric and self-harm adjusted for age, free school meal status, ethnic group. Model 3 is an adjusted model of each network metric and self-harm adjusted for age, free school meal status, ethnic group, and a network control variable (*a [adjusted for friendship group size], *b [popularity adjusted for sociality, sociality adjusted for popularity]

Girls only. *uOR* unadjusted odds ratio, *aOR* adjusted odds ratios

**Table 5:** Estimated Odds Ratios for the Effects of Sociometric Network Variables on Lifetime Self-harm among Boys Only

	Model 1		Model 2		Model 3*^ab^	
	uOR (95%CI)	*p*	aOR (95%CI)	*p*	aOR (95%CI)	*p*
Friends’ self-harm^*a^						
0 friends report self-harm	*Ref*		*Ref*		*Ref*	
At least 1 friend report self-harm	1.40 (0.94, 2.08)	0.094	1.27 (0.84, 1.91)	0.242	1.36 (0.88, 2.10)	0.156
Friends’ self-harm^*a^						
0 friends report self-harm	*Ref*		*Ref*		*Ref*	
1 friend reports self-harm	1.07 (0.67, 1.70)	0.765	0.96 (0.60, 1.56)	0.900	1.05 (0.64, 1.72)	0.843
2 friends report self-harm	1.96 (1.05, 3.64)	0.032	1.85 (0.98, 3.51)	0.057	2.13 (1.09, 4.16)	0.027
≥3 friends report self-harm	3.63 (1.54, 8.58)	0.003	3.23 (1.31, 7.94)	0.010	3.76 (1.48, 9.55)	0.005
Friendship-group density^*a^						
Medium density	*Ref*		*Ref*		*Ref*	
Low density	0.94 (0.63, 1.41)	0.784	0.87 (0.57, 1.32)	0.533	0.90 (0.58, 1.38)	0.635
High density	0.57 (0.23, 1.93)	0.461	0.65 (0.22, 1.90)	0.435	0.63 (0.21, 1.85)	0.403
Bridging^*a^	1.03 (1.00, 1.06)	0.009	1.03 (1.00, 1.06)	0.010	1.05 (1.01, 1.08)	0.002
Popularity^*b^	1.01 (0.93, 1.10)	0.708	1.02 (0.93, 1.11)	0.600	1.03 (0.94, 1.13)	0.467
Sociality^*b^	0.95 (0.85, 1.05)	0.365	0.94 (0.84, 1.05)	0.313	0.93 (0.84, 1.04)	0.259
Isolated						
No	*Ref*		*Ref*			
Yes	2.56 (1.36, 4.81)	0.004	2.69 (1.41, 5.11)	0.002	—	—
	n, 1026		n, 1026		n, 1026	

All models adjusted for clustering at the school level. Model 1 is an unadjusted bivariable model of each network metric with self-harm. Model 2 is an adjusted model of each network metric and self-harm adjusted for age, free school meal status, ethnic group. Model 3 is an adjusted model of each network metric and self-harm adjusted for age, free school meal status, ethnic group, and a network control variable (*a [adjusted for friendship group size], *b [popularity adjusted for sociality, sociality adjusted for popularity]

Boys only. *uOR* unadjusted odds ratio, *aOR* adjusted odds ratios

### Sensitivity Analyses

Analyses were repeated excluding participants from six networks that had >40% missing data at T1 and alternative specifications of sociometric variables (e.g., different cut points for friendship group density) were tested (Table S8). Overall, the findings were not substantively changed and the direction and size of effects remained similar.

## Discussion

Research and theory highlight the important role of peer-relationships for adolescent mental health, including self-harm, and a growing evidence base suggests peer-friendship networks shape adolescent mental health more broadly. Yet, limited research has empirically examined how both the composition and structure of in-school peer-friendship networks relate to early adolescent self-harm through a sociometric lens. In particular, how a comprehensive range of network metrics such as friends’ self-harm (composition) and measures of integration among peer-friendships (network structure) relate to self-harm, and how these processes differ by gender or across diverse, inner-city school settings, has remained unclear. This study addressed these gaps by exploring how sociometric in-school peer-friendship networks and gender relate to self-harm among youth from a contemporary, diverse inner-city London cohort. The findings suggest friends’ self-harm was associated with increased odds of adolescent’s own self-harm. In addition, both over- and under- integration in the wider peer-network was associated with increased odds of self-harm, whereas being part of a highly interconnected friendship group (high friendship group density) was protective. Effects were similar for boys and girls. The findings indicate that both peer-friendship network composition and structure matter for early adolescent self-harm regardless of gender.

### Network Composition

Strikingly, almost half of adolescents were connected to at least one friend that had reported self-harm, and there was strong evidence this was associated with increased odds of adolescents’ own self-harm. In addition, the association was cumulative: the greater the number of friends’ that reported self-harm, the greater the estimated odds of adolescent’s own reported self-harm, with a more than three-fold increase in odds for those with three or more friends who report self-harm. Importantly, the measure in this study was based on adolescents’ friends’ own self-report of behavior, indicating that irrespective of perception, peer-network composition (i.e., the behaviors of who youth are connected to in their peer-friendship networks) is an important factor that is associated with early adolescent self-harm. This finding aligns with prior research (Bilello, Townsend, Broome, Armstrong, et al., 2024) and with the few other sociometric studies that measured peer-network composition and self-harm in more limited ways (i.e., self-cutting only, or NSSI only) (Crudgington et al., 2023). Whilst specific mechanisms underlying the association were beyond the scope of the current study, findings align with broader theories and evidence, such as peer influence in adolescence, as the cumulative impact of having multiple friends who self-harm may increase opportunities for exposure, social learning, or reinforcement of the behavior as a coping mechanism (Nock, 2009).

### Network Structure

Self-harm was also associated with how adolescents were connected among their in-school peers. For example, sociality (sending friendship nominations) was associated with reduced odds of self-harm, in line with prior work that suggests seeing oneself as part of the school context and having friends to nominate may be protective for wellbeing (Copeland et al., 2019). Also consistent with prior research on network integration and mental health, this study found evidence that network metrics relating to over-integration in peer-friendship networks were associated with self-harm (Crudgington et al., 2023).

### Network Over-integration

The findings suggest that over-integration in the wider school year group (bridging, popularity) may be detrimental for self-harm, whereas integration in immediate friendship groups (high friendship group density) could be protective. High betweenness centrality (bridging) might reflect youth who have difficulty in forming tight-knit friendship groups which may lead to social stress, in line with prior research on bridging and mental distress (Carboni & Gilman, 2012) and self-cutting (Copeland et al., 2019). This theorized mechanism linking bridging to self-harm was reinforced by the YPCC, who reflected that being in a bridging position might be taxing on mental wellbeing and a “form of isolation” from those they are bridging. In addition, popularity may be associated with increased social pressure, which could contribute to emotional distress and increased odds of self-harm (Allen et al., 2005).

By contrast, being part of a high-density friendship group may buffer against stress or reflect social support that is more readily available and might protect against self-harm. Being part of a highly inter-connected friendship group may foster a sense of belonging above and beyond just seeing oneself as part of the wider school and having friends to name (e.g., sociality). Alternatively, “friends being friends with each other” may reflect experiencing less conflict among one’s peer-friendship group or reduced social strain which may be protective against distress, compared with youth who have disconnected friends (Bearman & Moody, 2004). More broadly, findings highlight the complexity of network integration among peers, where both close peer dynamics (friendship groups) and broader peer-group integration may play a role for adolescent self-harm. However, although these findings add to the limited evidence base on peer-network structure and self-harm, caution is emphasized with the interpretation of betweenness, popularity, and sociality as effects sizes were small.

### Network Under-integration

In addition, this study found that under-integration in peer-networks, specifically social isolation, matters for self-harm. Being isolated from peers (having zero or only one friend) was associated with an almost two-fold increase in the odds of self-harm, suggesting a lack of connection to peers may be more detrimental for self-harm than over-integration among peers. This also indicates that there may be something specific about isolation from a school year group, a developmentally important social group in early adolescence, that may increase the likelihood of self-harm, when prior work shows isolation from wider school networks matters for serious mental distress like suicide ideation and attempts (Bearman & Moody, 2004). While some adolescents may have meaningful friendships outside school, school-based peer integration has been shown to be highly consequential for adolescent mental health, even when accounting for relationships beyond the school context (Crosnoe, 2000). Indeed, sociometric isolation from school year groups may capture adolescents that have poor mental health that leads to withdrawal from same-aged peers or that self-harm may be related to adultifying behaviors that spur friendships outside of their school year (not captured here). Although the cross-sectional nature of this study means that it is not possible to ascertain temporality of effects, and underlying mechanisms are beyond this study’s scope, this is a novel and important contribution to the literature on self-harm and peers.

### Gender, Context, and Adolescent Development

In this study, there was no evidence that gender moderated any observed associations, which means that the effect of peer-networks on self-harm was similar for girls and boys. This finding is inconsistent with prior theorizing of the emotional costs and benefits of social networks differing for boys and girls, and with the few sociometric studies that explored networks, gender, and behaviors similar to self-harm (Crudgington et al., 2023; Rose & Rudolph, 2006). This contrasting finding may be reflective of self-harm and social networks in a contemporary, diverse, urban sample of inner-city adolescents. It also represents a novel contribution, as of the limited sociometric studies on peer-networks and self-harm and suicidal behavior, few have formally tested network-by-gender interactions in relation to self-harm.

However, it is possible that there are small or modest variations by gender that the sample size for this study was too small to detect with precision. Two findings in the gender stratified analyses are worthy of comment. First, among girls only there was evidence that having only one friend report self-harm was associated with increased odds of self-harm, whereas patterns were similar for both genders when two or more friends reported self-harm (i.e., odds of self-harm increased). This might suggest that among girls, the presence of even one friend reporting self-harm is associated with self-harm, but for boys, it is when multiple friends report self-harm that the estimated odds of self-harm increase. This disparity may reflect that girls might be more likely to disclose their self-harm behavior to friends compared with boys.

Second, boys were more isolated (7%) compared with girls (3%), and isolation was associated with an almost 3-fold increase in odds of self-harm among boys, whereas there was little effect among girls. This could reflect that girls and boys might respond differently to social isolation at school. This is also in line with prior literature that suggests boys tend to be more isolated than girls (Umberson et al., 2022) and that isolation in particular is associated with mental health problems among boys (Liu et al., 2020). However, any inferences based on differences observed here need to be made cautiously and further research is needed in larger samples to examine these variations.

Importantly, findings are based on adolescents aged 11 to 14, reflecting early adolescence - a period when self-harm typically first emerges (Gillies et al., 2018) and when peer relationships become especially salient. As such, peer-friendships are an important aspect of development to focus on during this stage of life, both to capture patterns in early adolescence (e.g., self-harm and where other mental health problems may first emerge) and also as a foundation for shaping later adolescent and long-term outcomes. The findings of this study suggest that peer-friendship networks matter for early adolescent self-harm, and further research is needed on whether peer-network effects persist into later adolescence or adulthood.

Additionally, this study is focused on a contemporary, diverse, inner-city sample of adolescents. Although some findings align with prior research, differences, such as the lack of gender differences, might highlight different social processes in contemporary inner-city London contexts. For example, there is a smaller gender difference in self-harm prevalence in this sample than typically observed in other UK samples (Morey et al., 2017; Patalay & Fitzsimons, 2021), suggesting that boys may face elevated risks here. Moreover, structural inequalities (e.g., socioeconomic disadvantage, racial and ethnic marginalization) may shape peer-friendship networks, which may influence both opportunities for social integration and the risks associated with different network positions for both boys and girls. The findings add to the limited evidence base on associations of peer-friendship networks and self-harm in diverse contexts.

### Limitations

There are a number of limitations that are important to consider. Self-harm was captured using a single item self-report measure validated in adolescent populations (Goodman, 2001), but it is a complex behavior that may vary in frequency, method, and severity in ways not captured here. Gender was measured via a binary self-report item of “girl or boy” which means that this study is unable to capture the full complexity of gender as a fluid construct. Adolescents’ friendship nominations were truncated at five, yet research suggests that youth nominate six friends on average (Neal, 2025), which means that the effect of over-integration in the network may be under-estimated in this study. In addition, some networks had a high proportion of missing data, and incomplete network data has the potential to bias network metrics. To mitigate this, measures more robust to missingness were used (e.g., based on both in- and out-degree), and key models were re-run on a subset of schools with more complete network data in sensitivity analyses that find consistent results, supporting the robustness of the findings.

This study is cross-sectional and used a lifetime measure of self-harm which means it is not possible to ascertain temporality of effects and adolescents could have self-harmed prior to starting secondary school. Although REACH is a longitudinal cohort study with sociometric data available across all waves, this analysis focused on the baseline data to allow for the initial construction, visualization, and analysis of the peer-friendship networks as a crucial first step. Analyses also incorporated adolescents aged 11 – 14 years of age which means results represent the average effects across different ages in the sample, but age-related variation of effects, even in early adolescence, may exist. Lastly, social networks may be culturally patterned and adjusting for ethnic group does not fully capture this complexity, and REACH is a localized study, thus findings might not generalize beyond this context.

### Implications

The findings of this study offer several important implications. Conceptually, this study suggests that early adolescent self-harm is a complex behaviour that may be influenced by peer-friendship networks among both girls and boys. Research should consider both peer-network composition (friend’s self-harm) and peer-network structure (both under- and over- integration) as findings indicate that adolescent self-harm is associated with different patterns of integration, with especially high risks from friends’ self-harm and social isolation. Practically, findings suggest the need for more than one approach to self-harm prevention in schools. Interventions might benefit from taking a more comprehensive peer-network focused approach for both genders. For example, identifying not only isolated youth but also considering how friendship group dynamics and the broader peer-context – including having multiple friends’ who self-harm - might increase self-harm risk for both girls and boys. Contextually, the involvement of the Young Person’s Community Champions (YPCC) strengthens the real-world relevance of these findings, reflecting the lived experiences of diverse adolescents in inner-city schools. This means that that results here should be considered as having realistic implications for interventions because they resonate with lived experience. The YPCC process also demonstrates the value of less extractive, more responsive research approaches with young people, which can enhance both interpretation of results relating to peer-networks and self-harm and their impact. Together, the results point to the need for peer-focused, network-informed, and inclusive strategies to address self-harm risk across varied adolescent social contexts.

### Future Research

The findings suggest key areas for future research. First, studies should utilize longitudinal network data to examine reciprocal dynamics between peer-friendship networks and self-harm and whether network metrics may have differential effects at different stages of adolescence. Second, future research should consider how social networks might relate to the frequency and/or severity of self-harm. Third, examining other intersections of identity with self-harm and networks beyond gender (e.g., sexual identity, ethnicity) could clarify how peer-networks may impact self-harm.

## Conclusion

Self-harm is a concerning health challenge in adolescence, a developmental period when peers shape health behaviors. Despite the theorized social functions of self-harm, few studies have examined self-harm through a social network lens. That is, how the composition and structure of sociometric in-school peer-friendship networks relate to self-harm, or whether these associations differ by gender remained unclear. This study addressed this gap by exploring how a comprehensive range of network metrics relate to self-harm among a diverse cohort of inner-city London youth, and potential gender differences in these associations. Having friends who reported self-harm was associated with higher odds of adolescents’ own self-harm, suggesting potential peer influence. Structural features of networks also mattered: both under-integration (isolation) and over-integration (popularity, betweenness) were associated with increased odds of self-harm, while sociality and friendship group density were associated with reduced odds. Associations were similar for boys and girls. The findings highlight that both who adolescents are friends with and how they are embedded in their peer-networks are important for understanding early adolescent self-harm, across genders.

## Supplementary information

Supporting Information
