# Supplementary material for: Peer-friendship Networks and Self-harm among Adolescents from Inner-city Schools: A Social Network Study
Source: J Youth Adolesc. 2025 Oct 3;55(1):152–67. doi: 10.1007/s10964-025-02264-y (PMC12815966; doi:10.1007/s10964-025-02264-y)
Supplement: Supplementary file 1 — Supporting Information [file 10964_2025_2264_MOESM1_ESM.docx]

**Supporting Information**

# **Manuscript Title: Peer-Friendship Networks and Self-harm Among Adolescents from Inner-City Schools: A Social Network Study**

**Authors:** Holly Crudgington, Rachel Blakey, Molly Copeland, Charlotte Gayer-Anderson, Samantha Davis, Katie Lowis, Esther Putzgruber, Thai-sha Richards, Jonas Kitisu, Adna Hashi, Karima Shyan Clement-Gbede, Niiokani Tettey, Daniel Stanyon, Alice Turner, Lynsey Dorn, Seeromanie Harding, Kamaldeep Bhui, Vanessa Pinfold, Gemma Knowles, Craig Morgan.

**S1. Correlations between network variables**

**S2. Network descriptives table**

**S3. Further information about sociometric variables**

**S4. Sociograms of all networks**

**S5. Missing data information**

**S6. Network characteristics by cohort and ethnic group**

**S7. Gender*network metric interaction results for lifetime self-harm**

**S8. Sensitivity analyses**

**S9. Full models with all covariate results for network metrics and self-harm**

**S10. Young Person Community Champion (YPCC) views**

**S1. Correlations between network variables**

|  | **1** | **2** | **3** | **4** | **5** |
| --- | --- | --- | --- | --- | --- |
| **Betweenness centrality (1)** | 1 |  |  |  |  |
| **In-degree (2)** | 0.39* | 1 |  |  |  |
| **Out-degree (3)** | 0.28* | 0.22* | 1 |  |  |
| **Total degree (4)** | 0.41* | 0.75* | 0.67* | 1 |  |
| **Ego-network density (5)** | -0.21* | 0.04 | -0.07* | -0.27* | 1 |
| n, 2203, *p <.001 | | | | | |

**S2. T1 Network descriptives table**

| **School** | **Year group friendship network** | **# of nodes at T1** | **Overall density** | **% of people missing on the friendship network questions *a** |  |
| --- | --- | --- | --- | --- | --- |
|  |  |  |  |  |  |
| 4 | Network 1 (Cohort 1) | 141 | 0.03 | 29.8 |  |
| 4 | Network 2 (Cohort 2) | 133 | 0.03 | 21.1 |  |
| 4 | Network 3 (Cohort 3) | 142 | 0.03 | 21.1 |  |
| 5 | Network 4 (Cohort 1) | 126 | 0.04 | 38.1 |  |
| 5 | Network 5 (Cohort 2) | 125 | 0.04 | 39.2 |  |
| 5 | Network 6 (Cohort 3) | 123 | 0.04 | 29.0 |  |
| 6 | Network 7 (Cohort 1) | 141 | 0.02 | 46.8 |  |
| 6 | Network 8 (Cohort 2) | 146 | 0.03 | 43.8 |  |
| 6 | Network 9 (Cohort 3) | 149 | 0.04 | 38.3 |  |
| 7 | Network 10 (Cohort 1) | 170 | 0.03 | 16.5 |  |
| 7 | Network 11 (Cohort 2) | 155 | 0.03 | 22.6 |  |
| 7 | Network 12 (Cohort 3) | 138 | 0.03 | 24.6 |  |
| 8 | Network 13 (Cohort 1) | 130 | 0.04 | 28.5 |  |
| 8 | Network 14 (Cohort 2) | 123 | 0.04 | 24.4 |  |
| 8 | Network 15 (Cohort 3) | 119 | 0.04 | 44.5 |  |
| 9 | Network 16 (Cohort 1) | 121 | 0.04 | 24.8 |  |
| 10 | Network 17 (Cohort 1) | 85 | 0.06 | 52.9 |  |
| 10 | Network 18 (Cohort 2) | 86 | 0.06 | 20.9 |  |
| 10 | Network 19 (Cohort 3) | 69 | 0.07 | 49.3 |  |
| 11 | Network 20 (Cohort 1) | 142 | 0.03 | 24.7 |  |
| 11 | Network 21 (Cohort 2) | 135 | 0.04 | 16.3 |  |
| 11 | Network 22 (Cohort 3) | 136 | 0.04 | 34.6 |  |
| 12 | Network 23 (Cohort 1) | 182 | 0.03 | 36.6 |  |
| 12 | Network 24 (Cohort 2) | 181 | 0.03 | 48.6 |  |
| 12 | Network 25 (Cohort 3) | 180 | 0.03 | 23.3 |  |

Cohort 1 = baseline year 7, Cohort 2 = baseline year 8, Cohort 3 = baseline year 9. T1 = time 1. *a = this is the percentage of missing friendship nomination data [of the nodes listed in the number of nodes per time point column], irrespective of participation status i.e., includes item non-response as well as pupil absence and non-participation.

**S3. Further information about sociometric variables**To accurately reflect out-degree 0 (i.e., adolescents who did not nominate friends but were present on the day of data collection), we conducted additional data cleaning. This involved manually inspecting responses to determine if adolescents with an out-degree of 0 (n, 1076) had completed the questions immediately following the friendship network section. There were 301 students who had an out-degree 0 who answered subsequent questions after the friendship nomination section. Their out-degree value was coded as 0, while the other participants were treated as missing (n,775).

**Betweenness centrality**The formula for betweenness centrality is given by shortest path betweenness of a vertex [***v***]:

$$C_{B}(v)=\sum_{i,j:i\neq j,i\neq v,j\neq v} \frac{g_{ivj}}{g_{ij}}$$

Where $g_{ivj}$ is the number of geodesics from *i* to *j* through *v*. Betweenness centrality values can be very large dependent on network size, because nodes have more opportunity to lie on the shortest path between others in a large network (i.e., in this dataset betweenness values range from 0 – 5880 and the variable is positively skewed). To aid interpretation in analyses, each adolescent’s betweenness score was divided by 100.

**Friendship group density (Ego-network density)**Ego-network density is the ratio of actual friendship ties among an adolescent’s send and receive network to all possible ties in their send and receive network, producing a statistic that ranges from 0 (no connections) to 1 (completely connected ego-network). The measure of ego-network density computed in igraph includes ego in the calculation. Whether a density value should be considered high, medium, or low depends on context. For example, an adolescent who belongs in an ego-network with 5 friends but with a density of 0-0.3 could be considered very low – as we tend to see higher density ties for positive ties and for ego-networks in general. However, there is no consensus in the field of Social Network Analysis (SNA) as to exactly what cut points should be used to reflect Low, Medium, and High. We defined low density as scoring 0-0.3, medium density as 0.31 – 0.59, and high density as 0.6-1. We explore different cut-points for density in sensitivity analyses (S8).

**S4. Sociograms of all networks**

**
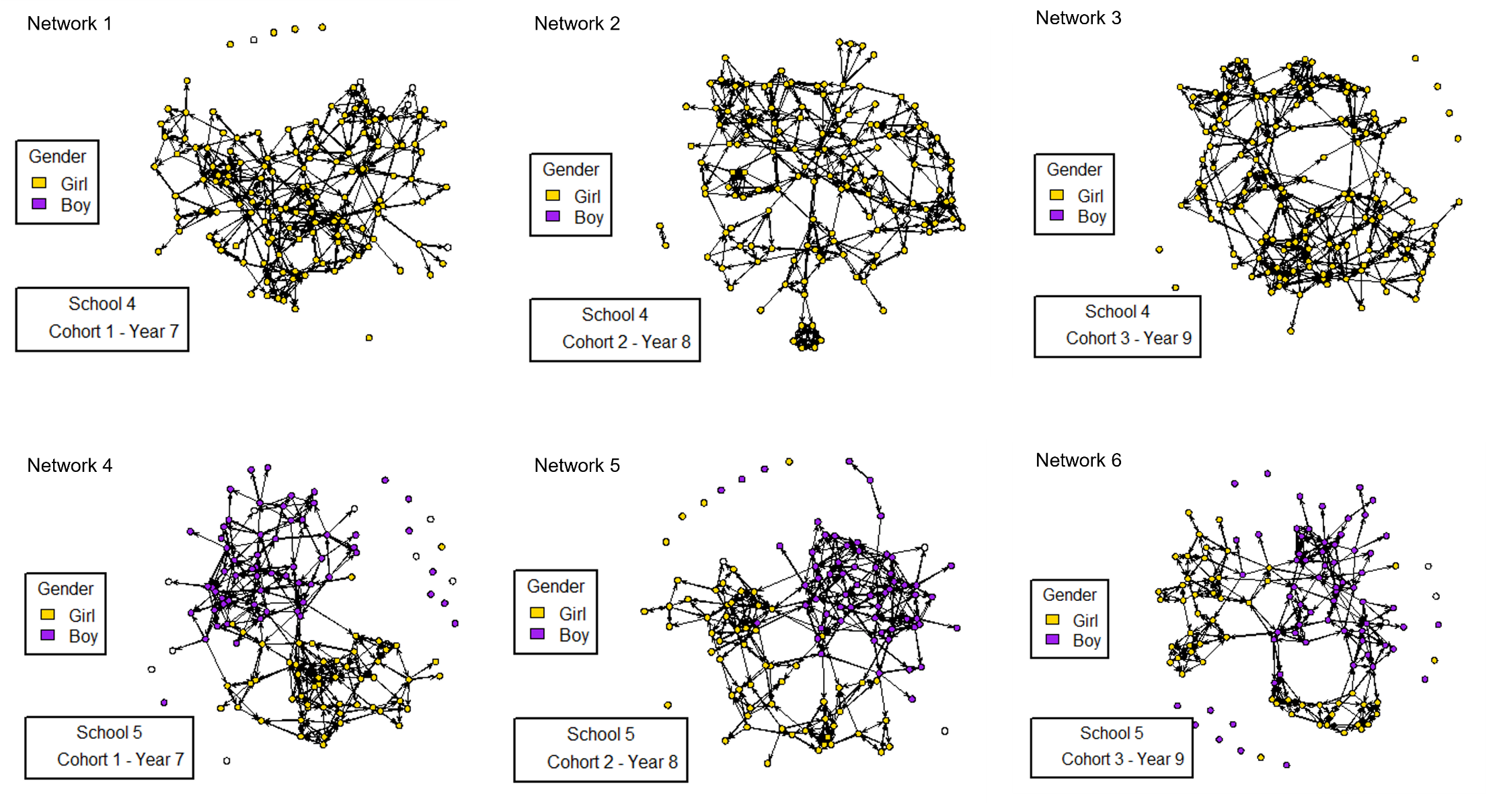
Sociograms of Schools 4 - 5, Networks #1 - 6**

**Sociograms of Schools 6 – 7, networks #7 to 12**

**
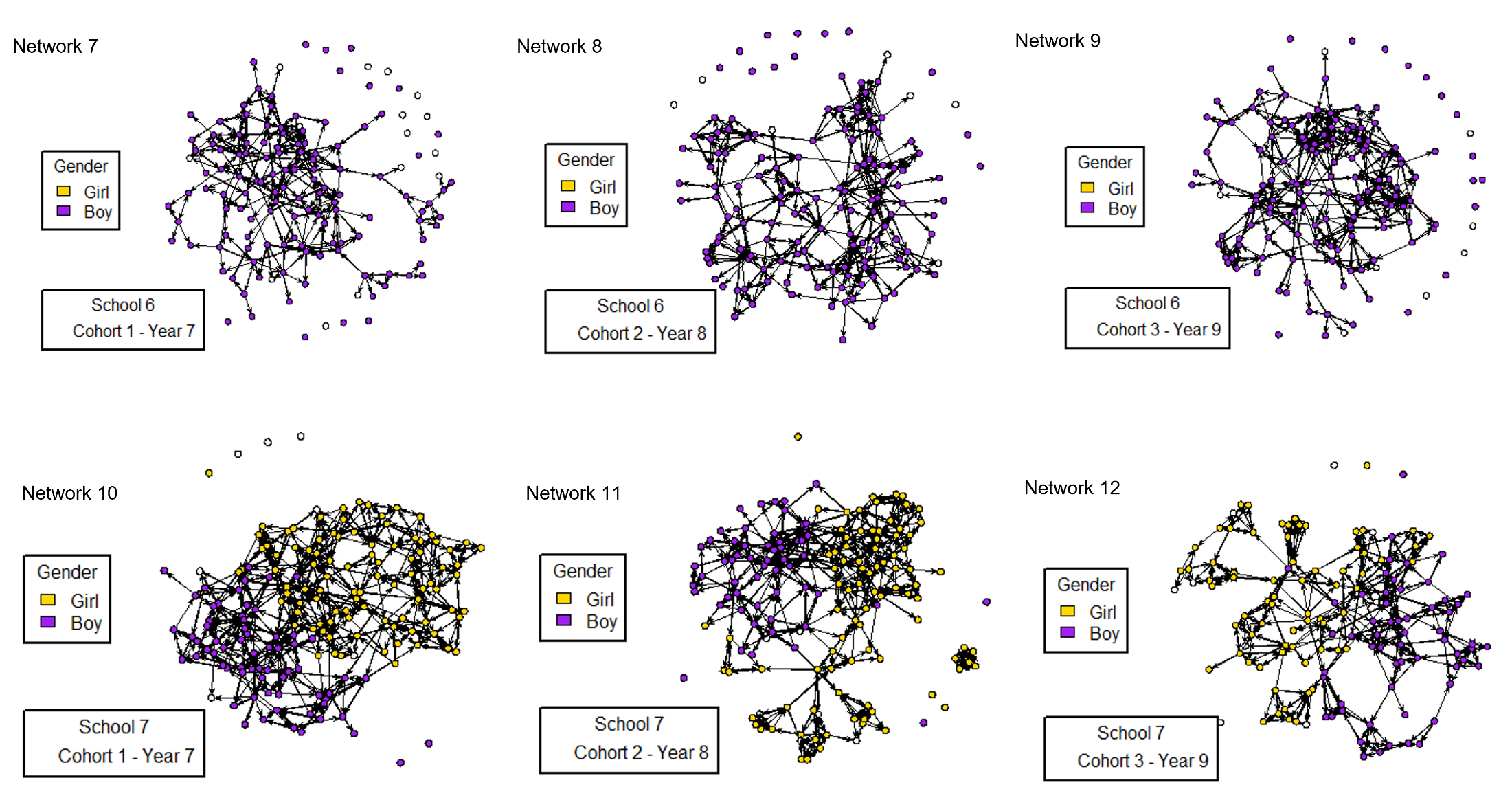
**

**Sociograms of Schools 8-9, Networks #13 - 16**


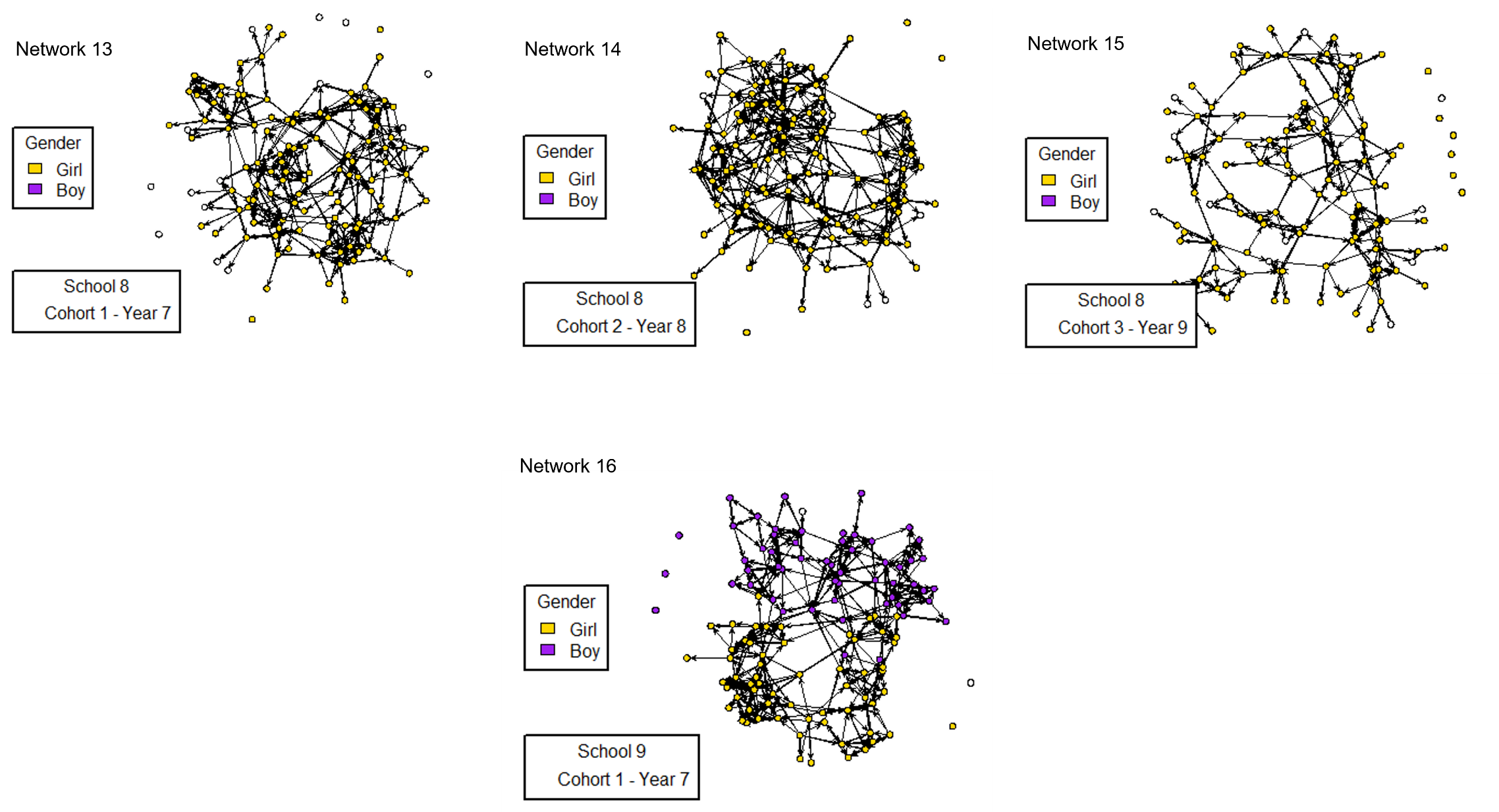


**Sociograms of Schools 10 – 11, Networks #17 – 22**


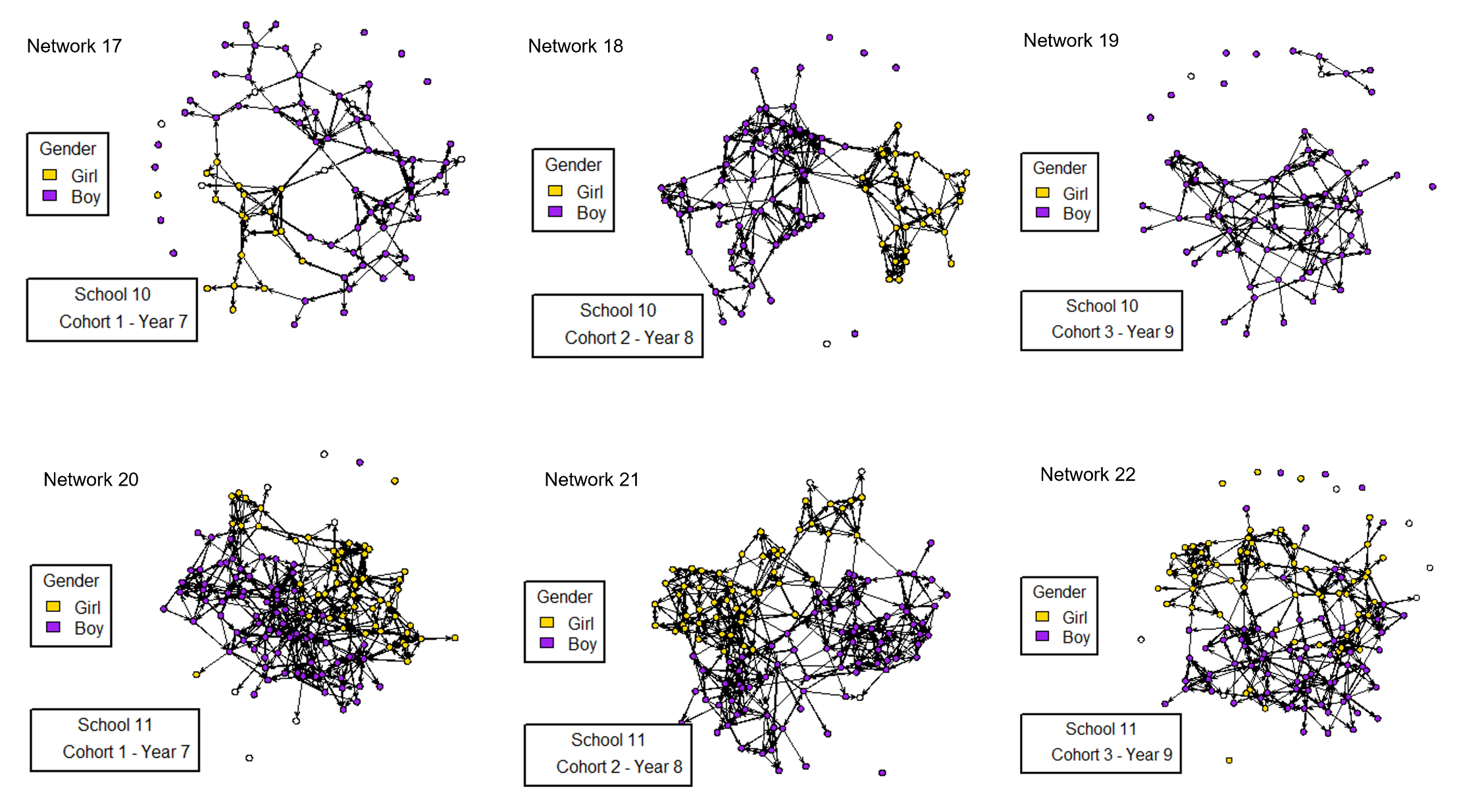


**Sociograms of School 12, networks #23 - 25**


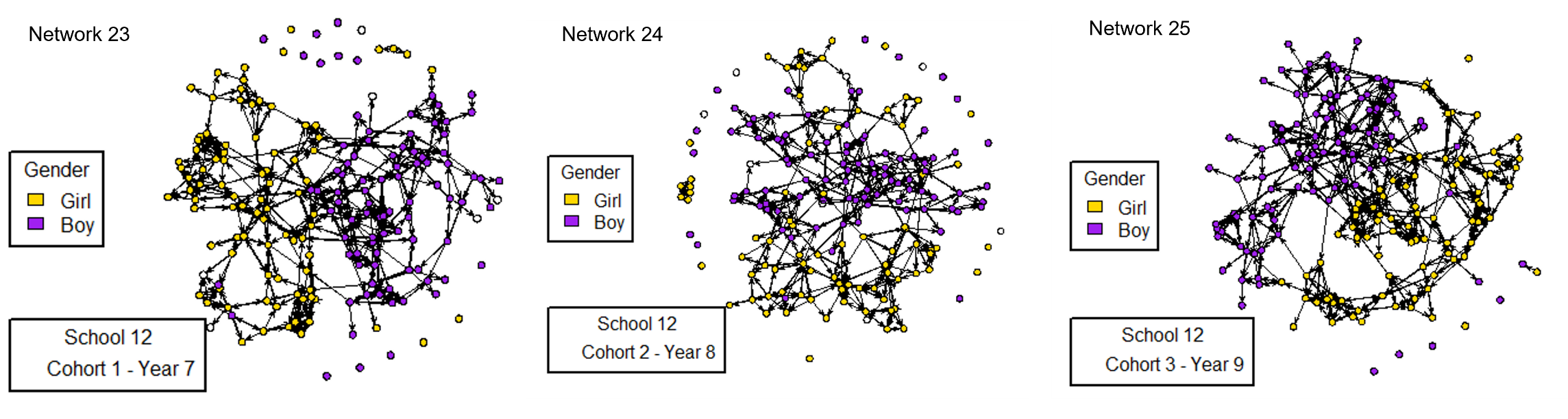


Network visualisations reflect everyone that was eligible to be part of a network at baseline irrespective of participation status.

**S5. Missing data information**

**S5.1. Comparison of analysis sample with the REACH baseline sample and those eligible to be in the networks at T1**

|  | **Analysis sample  (complete cases)**  **n (2, 2203)** | | **Total ____ baseline sample - eligible to be in a network at T1 (9 schools, 25 networks) *a**  **n (3, 378)** | | **Total ____ baseline sample - participated (12 schools) *b**  **n (4, 353)** | |
| --- | --- | --- | --- | --- | --- | --- |
|  |  |  |  |  |  |  |
| **Socio-demographics** | **n** | **%** | **n** | **%** | **n** | **%** |
| **Gender** | **n (2, 203)** | | **n (3, 193)** | | **n (4,353)** | |
| Boys | 1,026 | 46.6 | 1,513 | 47.4 | 2,140 | 49.2 |
| Girls | 1,177 | 53.4 | 1,680 | 52.6 | 2,213 | 50.8 |
| **Year Group** | **N (2, 203)** | | **n (3, 378)** | | **n (4,353)** | |
| Year 7 (@ baseline) | 733 | 33.3 | 733 | 33.3 | 1,593 | 36.6 |
| Year 8 (@ baseline) | 720 | 32.7 | 720 | 32.7 | 1,423 | 32.7 |
| Year 9 (@ baseline) | 750 | 34.0 | 750 | 34.0 | 1,337 | 30.7 |
| **Ethnic group** | **n (2, 203)** | | **n (3, 187)** | | **n (4,353)** | |
| White British | 372 | 16.9 | 520 | 16.3 | 639 | 14.7 |
| Indian, Pakistani, Bangladeshi | 112 | 5.1 | 146 | 4.6 | 180 | 4.1 |
| Black Caribbean | 291 | 13.2 | 435 | 13.7 | 71 | 16.4 |
| Other Black | 68 | 3.1 | 100 | 3.1 | 130 | 3.0 |
| Mixed/multiple | 129 | 5.9 | 186 | 5.8 | 243 | 5.6 |
| Any Other | 145 | 6.6 | 203 | 6.4 | 299 | 6.9 |
| Black African | 639 | 29.0 | 915 | 28.7 | 1,112 | 25.6 |
| Non-British White | 174 | 7.9 | 256 | 8.0 | 430 | 9.9 |
| Mixed White and Black | 188 | 8.5 | 288 | 9.0 | 386 | 8.9 |
| Latin American | 85 | 3.9 | 138 | 4.3 | 220 | 5.1 |
| **Free School Meals** | **n (2, 203)** | | **n (2, 803) *c** | | **n (4, 113)** | |
| No | 1,775 | 80.6 | 2,244 | 80.0 | 3,136 | 76.3 |
| Yes | 428 | 19.4 | 559 | 20.0 | 977 | 23.8 |
| **Lifetime self-harm** | **n (2, 203)** | | **n (2, 562) *d** | | **n (3, 061)** | |
| No | 1,902 | 86.3 | 2,209 | 86.2 | 2,620 | 85.6 |
| Yes, ever | 301 | 13.7 | 353 | 13.8 | 441 | 14.4 |

*a = The eligible REACH sample at baseline was 4,945. Three schools were excluded from these analyses culminating in 25 separate year groups (networks) across 9 schools, with 3378 participants that were eligible to be in one of those networks.
*b = There were 4945 adolescents who were eligible to be part of the REACH baseline sample from all 12 schools (i.e., including the pilot schools). Of the 4945 eligible, 4,353 participated in the REACH study. The reason for non-participation from pupils included school absenteeism (n= 353, 7%), parental opt-out (n, 167, 3%), non-assent (n = 57, 1%), technical issues (n = 15, 0.3%).
*c = reasons for missing data on free-school meals - 409 did not participate (reasons [opt out n= 125], [non-assent n=33], [absent n = 241], [leaver/move [n =2], [technical issues n = 8], of those that did participate (2, 969) reasons for missing data were skipped/didn't reach that part of the questionnaire (n = 6) don't know (n = 145), refused (n = 15).
*d = reasons for missing data on self-harm - 409 did not participate (reasons [opt out n= 125], [non-assent n=33], [absent n = 241], [leaver/move [n =2], [technical issues n = 8. Of those that did participate (2969) - reasons for missing data included skipped/didn't reach that part of the questionnaire (n = 93), refused (n= 314).

**S5.2. Analysis sample compared with those who were not in the analysis sample but were eligible to be part of the baseline networks (categorical variables)**

| **Total eligible  (9 schools - 25 networks) n = 3378*a** | **Analysis sample (n = 2203)** | **Not included in  analysis sample (n = 1175) *b** | **Chi square** |
| --- | --- | --- | --- |
|  | **n (%)** | **n (%)** | **χ2(df)*p*** |
| **Gender (n, 3193)** |  |  | 1.87 (1) .170 |
| Boys | 1026 (46.57) | 487 (49.19) |  |
| Girls | 1177 (53.43) | 503 (50.81) |  |
| **Year Group (n, 3378)** |  |  | 36.95 (2) <.001 |
| Year 7 (@ baseline) | 505 (42.98) | 505 (42.98) |  |
| Year 8 (@ baseline) | 367 (31.23) | 367 (31.23) |  |
| Year 9 (@ baseline) | 303 (25.79) | 303 (25.79) |  |
| **Ethnic group (n, 3187)** |  |  | 12.97 (9) .164 |
| White British | 372 (16.89) | 148 (15.04) |  |
| Indian, Pakistani, Bangladeshi | 112 (5.08) | 34 (3.46) |  |
| Black Caribbean | 291 (13.21) | 144 (14.63) |  |
| Other Black | 68 (3.09) | 32 (3.25) |  |
| Mixed/multiple | 129 (5.86) | 57 (5.79) |  |
| Any Other | 145 (6.58) | 58 (5.89) |  |
| Black African | 639 (29.01) | 276 (28.05) |  |
| Non-British White | 174 (7.90) | 82 (8.33) |  |
| Mixed White and Black | 188 (8.53) | 100 (10.16) |  |
| Latin American | 85 (3.86) | 53 (5.39) |  |
| **Free School Meals (n, 2803)** |  |  | 1.70 (1) .191 |
| No | 1775 (80.57) | 469 (78.17) |  |
| Yes | 428 (19.43) | 131 (21.83) |  |

*a = Eligible adolescents from 25 networks (year groups) across 9 schools at T1.

*b = 1175 people were not included in our analysis sample. Of those, 766 (65.19%) participated in REACH but were missing data on a variable used in this analysis. 409 (34.81%) did not participate due to the following reasons: parental opt-out (n = 125, 10.64%), non-assent (n = 33, 2.81%), leaver/mover (n = 2, 0.17%), absence (n = 241, 20.51%), technical issues (n =8, 0.68%).

**S6. Network characteristics by cohort and ethnic group**

The focus of the main manuscript is on self-harm, social networks and gender, but these descriptives are provided for information and completeness. In the absence of a priori hypotheses regarding age and ethnic group (and small cell sizes), we do not make any inferences based solely on the descriptive data here.

**Table S6.1. Network characteristics by cohort (year group)**

| **Network Metrics** | **Total**  **(n = 2203)** | **Cohort 1 Year 7**  **(n = 733)** | **Cohort 2**  **Year 8**  **(n = 720)** | **Cohort 3**  **Year 9**  **(n = 750)** |
| --- | --- | --- | --- | --- |
| **Friends’ self-harm, n(%)** |  |  |  |  |
| 0 friends report self-harm | 1215 (55.2) | 450 (61.4) | 356 (49.4) | 409 (54.5) |
| 1 friend reports self-harm | 674 (30.6) | 193 (26.3) | 244 (33.9) | 237 (3.6) |
| 2 friends report self-harm | 230 (10.4) | 72 (9.8) | 85 (11.8) | 73 (9.7) |
| ≥3 friends report self-harm | 84 (3.8) | 18 (2.5) | 35 (4.9) | 31 (4.1) |
| **Isolated, n(%)** |  |  |  |  |
| No | 2097 (95.2) | 713 (97.3) | 686 (95.3) | 698 (93.1) |
| Yes | 106 (4.8) | 20 (2.7) | 34 (4.7) | 52 (6.9) |
| **Popularity (M±S.D)** | 3.4 (2.2) | 3.5 (2.3) | 3.3 (2.2) | 3.3 (2.1) |
| **Sociality (M±S.D)** | 4.0 (1.7) | 4.2 (1.5) | 4.1 (1.6) | 3.8 (1.9) |
| **Betweenness centrality/100, (M±S.D)*** | 4.2 (6.2) | 4.56 (5.6) | 4.11 (6.2) | 3.95 (6.7) |
| **Friendship group density n(%)** |  |  |  |  |
| Low density (0 - 0.3) | 798 (36.2) | 294 (40.1) | 263 (36.5) | 241 (32.1) |
| Medium density (0.31 - 0.59) | 1189 (54.0) | 396 (54.0) | 375 (52.1) | 418 (55.7) |
| High density (0.6 - 1) | 216 (9.8) | 43 (5.9) | 82 (11.4) | 91 (12.1) |
| **Friendship group size (M+SD)** | 5.6 (2.1) | 5.8 (2.1) | 5.58 (2.1) | 5.3 (2.2) |

M = mean, S.D standard deviation, n = number. Where necessary, small cell sizes have been omitted for data protection.

**Table S6.2. Network characteristics by ethnic group**

| **Network Metrics** | **Any other**  **(n, 145)** | **Black African**  **(n, 639)** | **Black Carib.**  **(n, 291)** | **Indian, Pakistani, Bangladeshi**  **(n, 112)** | **Latin A.**  **(n, 85)** | **Mixed White & Bl (n, 188)** | **Mixed/Mult.**  **(n, 129)** | **Non-British White**  **(n, 174)** | **Other Black**  **(n, 68)** | **White British**  **(n, 372)** |
| --- | --- | --- | --- | --- | --- | --- | --- | --- | --- | --- |
| **Friends’ self-harm n(%)** |  |  |  |  |  |  |  |  |  |  |
| 0 friends report self-harm | 75 (51.7) | 375 (58.7) | 133 (45.7) | 80 (71.4) | 37 (43.5) | 97 (51.6) | 77 (59.7) | 95 (54.6) | 40 (58.8) | 206 (55.4) |
| At least 1 friend reports self-harm | 70 (48.3) | 264 (41.3) | 158 (54.3) | 32 (28.6) | 48 (56.5) | 91 (48.4) | 52 (40.3) | 79 (45.4) | 28 (41.2) | 166 (44.6) |
| **Popularity (M±S.D)** | 3.04 (2.0) | 3.37 (2.2) | 3.31 (2.1) | 2.85 (2.3) | 3.07 (2.2) | 3.51 (2.4) | 3.31 (2.1) | 3.25 (2.0) | 3.05 (2.0) | 3.71 (2.3) |
| **Sociality (M±S.D)** | 3.99 (1.7) | 3.86 (1.9) | 4.07 (1.7) | 3.88 (1.7) | 4.07 (1.5) | 4.23 (1.5) | 4.11 (1.5) | 4.08 (1.6) | 3.91 (1.9) | 4.29 (1.4) |
| **Bridging, (M±S.D)*** | 2.98 (4.6) | 3.16 (4.7) | 4.86 (6.6) | 3.41 (5.4) | 4.50 (6.3) | 5.37 (7.0) | 4.78 (7.2) | 3.97 (6.0) | 3.31 (4.7) | 5.58 (7.7) |
| **Friendship group size (M+SD)** | 5.28 (2.0) | 5.55 (2.3) | 5.93 (2.2) | 4.78 (1.6) | 5.4 (2.1) | 5.80 (2.2) | 5.47 (2.1) | 5.44 (1.7) | 5.22 (2.2) | 5.74 (2.0) |

M = mean, S.D standard deviation, n = number, I P D = Indian, Pakistani, Bangladeshi. Where necessary, network metrics that had small groups (cell sizes) have been omitted for data protection

**S7.** **Gender × network metric interaction results for lifetime self-harm**

|  | **Overall**^*a^ | **Boys^*b^** | **Girls^*b^** | **p value for  gender * network metric interaction** |
| --- | --- | --- | --- | --- |
|  | **aOR (95%CI) *p*** | **aOR (95%CI) *p*** | **aOR (95%CI) *p*** |  |
| **Friends' self-harm** |  |  |  |  |
| 0 friends report self-harm | *Ref* | *Ref* | *Ref* |  |
| At least one friend reports self-harm | 1.63 (1.25, 2.13), <.001 | 1.32 (0.87, 2.00), 0.176 | 1.86 (1.33, 2.61), <.001 | 0.194 |
| **Friends' self-harm** |  |  |  |  |
| 0 friends report self-harm | *Ref* | *Ref* | *Ref* | 0.666 |
| 1 friend reports self-harm | 1.32 (0.98, 1.78), 0.064 | 1.06 (0.66, 1.70), 0.804 | 1.52 (1.04, 2.20), 0.028 |  |
| 2 friends report self-harm | 2.35 (1.60, 3.46), <.001 | 1.96 (1.03, 3.70), 0.039 | 2.62 (1.63, 4.22), <.001 |  |
| >3 friends report self-harm | 3.72 (2.20, 6.28), <.001 | 3.51 (1.45, 8.48), 0.005 | 3.92 (2.07, 7.39), <.001 |  |
| **Friendship group density** |  |  |  |  |
| Medium Density | *Ref* | *Ref* | *Ref* | 0.841 |
| Low Density | 0.93 (0.70, 1.23), 0.639 | 0.89 (0.59, 1.35), 0.608 | 0.97 (0.67, 1.40), 0.878 |  |
| High Density | 0.51 (0.31, 0.85), 0.010 | 0.64 (0.22, 1.89), 0.428 | 0.49 (0.27, 0.86), 0.014 |  |
| **Bridging** | 1.02 (1.00, 1.05), 0.014 | 1.03 (1.00, 1.06), 0.011 | 1.01 (0.99, 1.04), 0.180 |  |
| **Popularity** | 1.05 (0.99, 1.12), 0.051 | 1.03 (0.94, 1.12), 0.528 | 1.08 (1.00, 1.17), 0.038 |  |
| **Sociality** | 0.95 (0.88, 1.02), 0.192 | 0.94 (0.84, 1.05), 0.266 | 0.96 (0.87, 1.06), 0.438 |  |
| **Isolated** |  |  |  |  |
| No | *Ref* | *Ref* | *Ref* |  |
| Yes | 1.73 (1.05, 2.83) 0.029 | 2.55 (1.37, 4.77), 0.003 | 1.14 (0.48, 2.68) 0.767 | 0.134 |
| **n, 2203** |  |  |  |  |

***a** Overall model estimates are the same as the final column of the main results table in the main manuscript (i.e., adjusted for gender, age, free-school meals, ethnic group, and a network control [where applicable]). ***b** Linear combination of estimates was used to extract gender-specific ORs directly from the interaction model that contained all control variables. Unadjusted interaction models showed similar patterns with no significant gender moderation (i.e., all interaction p values >.05)

**S8. Sensitivity analyses**

**S8.1 Estimated odds ratios for the effects of friendship group density (different cut points of the friendship group density variable) and lifetime self-harm among adolescents overall**

|  | **Model 1** | | **Model 2** | | **Model 3** | |
| --- | --- | --- | --- | --- | --- | --- |
|  | **uOR (95%CI)** | ***p*** | **aOR (95%CI)** | ***p*** | **aOR (95%CI)** | ***p*** |
| **Friendship group density^*a^** |  |  |  |  |  |  |
| Medium density (0.30 - 0.49) | *Ref* |  | *Ref* |  | *Ref* |  |
| Low density (0 - 0.29) | 0.90 (0.68, 1.20) | 0.492 | 0.99 (0.74, 1.33) | 0.982 | 0.97 (0.72, 1.31) | 0.876 |
| High density (0.5 - 1) | 0.77 (0.54, 1.11) | 0.169 | 0.68 (0.47, 0.99) | 0.045 | 0.70 (0.48, 1.01) | 0.058 |
| **Ego-network density^*a^** |  |  |  |  |  |  |
| Medium density (0.35 - 0.59) | *Ref* |  | *Ref* |  | *Ref* |  |
| Low density (0 - 0.34) | 0.85 (0.66, 1.10) | 0.220 | 0.93 (0.71, 1.21) | 0.596 | 0.91 (0.70, 1.19) | 0.531 |
| High density (0.61 - 1) | 0.58 (0.35, 0.95) | 0.032 | 0.49 (0.30, 0.82) | 0.007 | 0.50 (0.30, 0.84) | 0.009 |
|  | n, 2203 | | n, 2203 | | n, 2203 | |

uOR = Unadjusted Odds Ratio, aOR = Adjusted Odds Ratios. All models adjusted for clustering at the school level.
Model 1 is an unadjusted univariable model assessing the association between friendship group density with self-harm. Model 2 is adjusted for gender, age, free school meal status, and ethnic group. Model 3 is further adjusted for friendship group size.

**S8.2. Estimated odds ratios for the effects of network metrics and lifetime self-harm among adolescents overall (on networks with <40% missing data)**

|  | **Model 1** | | **Model 2** | | **Model 3*^ab^** | |
| --- | --- | --- | --- | --- | --- | --- |
|  | **uOR (95%CI)** | **p** | **aOR (95%CI)** | **p** | **aOR (95%CI)** | **p** |
| **Friends’ self-harm ^*a^** |  |  |  |  |  |  |
| 0 friends report self-harm | *Ref* |  | *Ref* |  | *Ref* |  |
| At least 1 friend reports self-harm | 1.70 (1.30, 2.24) | <.001 | 1.63 (1.23, 2.16) | 0.001 | 1.61 (1.20, 2.16) | 0.001 |
| **Friends’ self-harm ^*a^** |  |  |  |  |  |  |
| 0 friends report self-harm | *Ref* |  | *Ref* |  | *Ref* |  |
| 1 friend reports self-harm | 1.33 (0.97, 1.81) | 0.074 | 1.25 (0.91, 1.73) | 0.157 | 1.27 (0.91, 1.76) | 0.145 |
| 2 friends report self-harm | 2.32 (1.57, 3.43) | <.001 | 2.31 (1.55, 3.43) | <.001 | 2.36 (1.56, 3.58) | <.001 |
| ≥ 3 friends report self-harm | 3.63 (2.13, 6.19) | <.001 | 3.51 (2.03, 6.05) | <.001 | 3.60 (2.05, 6.34) | <.001 |
|  |  |  |  |  |  |  |
| **Friendship-group density^*a^** |  |  |  |  |  |  |
| Medium density | *Ref* |  | *Ref* |  | *Ref* |  |
| Low density | 0.92 (0.69, 1.23) | 0.614 | 1.06 (0.78, 1.43) | 0.692 | 1.04 (0.77, 1.41) | 0.775 |
| High density | 0.52 (0.30, 0.91) | 0.022 | 0.46 (0.26, 0.80) | 0.006 | 0.47 (0.26, 0.82) | 0.009 |
| **Bridging^*a^** | 1.02 (1.00, 1.04) | 0.024 | 1.02 (1.00, 1.04) | 0.016 | 1.02 (1.00, 1.04) | 0.039 |
| **Popularity^*b^** | 1.05 (0.99, 1.11) | 0.085 | 1.05 (0.99, 1.12) | 0.066 | 1.06 (1.00, 1.13) | 0.044 |
| **Sociality^*b^** | 0.97 (0.89, 1.05) | 0.499 | 0.97 (0.89, 1.05) | 0.505 | 0.95 (0.87, 1.04) | 0.290 |
| **Isolated** |  |  |  |  |  |  |
| No | *Ref* |  | *Ref* |  |  |  |
| Yes | 2.12 (1.18, 3.83) | 0.012 | 2.38 (1.29, 4.38) | 0.005 | - | - |
|  | n, 1785 | | n, 1785 | | n, 1785 | |

uOR = Unadjusted Odds Ratio, aOR = Adjusted Odds Ratios. All models adjusted for clustering at the school level.  
Model 1 is an unadjusted univariable model assessing the association between each network metric with self-harm. Model 2 is adjusted for gender, age, free school meal status, and ethnic group. Model 3 is further adjusted for a network control variable (*a [adjusted for friendship group size], *b [popularity adjusted for sociality, sociality adjusted for popularity].

**S9. Full models with all covariate results for network metrics and self-harm**

**Table S9.1 Estimated odds ratios for the effects of friends’ self-harm and lifetime self-harm among adolescents overall**

|  | **Model 1** | | | **Model 2** | | **Model 3** | |
| --- | --- | --- | --- | --- | --- | --- | --- |
|  | **uOR (95%CI)** | | ***p*** | **aOR (95%CI)** | ***p*** | **aOR (95%CI)** | ***p*** |
| **Friend's self-harm** |  | |  |  |  |  |  |
| 0 friends report self-harm | ***Ref*** | |  | ***Ref*** |  | ***Ref*** |  |
| 1 friend reports self-harm | 1.37 (1.03 - 1.82) | | 0.029 | 1.29 (0.96 - 1.72) | 0.083 | 1.32 (0.98 - 1.78) | 0.064 |
| 2 friends report self-harm | 2.30 (1.58 - 3.32) | | <.001 | 2.24 (1.55 - 3.24) | <.001 | 2.35 (1.60 - 3.46) | <0.001 |
| ≥3 friends report self-harm | 3.86 (2.31 - 6.44) | | <.001 | 3.53 (2.13 – 5.85) | <.001 | 3.72 (2.20 - 6.28) | <0.001 |
| **Adolescent's characteristics** |  | |  |  |  |  |  |
| **Gender** |  |  | |  |  |  |  |
| Boy |  | |  | ***Ref*** |  | ***Ref*** |  |
| Girl |  | |  | 1.54 (1.19 - 2.00) | 0.001 | 1.53 (1.18 - 1.99) | 0.001 |
| **Age** |  | |  | 1.21 (1.06 – 1.39) | 0.003 | 1.21 (1.06 - 1.38) | 0.004 |
| **Ethnicity** |  | |  |  |  |  |  |
| White British |  | |  | ***Ref*** |  | ***Ref*** |  |
| Indian, Pakistani, Bangladeshi |  | |  | 0.93 (0.49 – 1.77) | 0.832 | 0.91 (0.48 – 1.74) | 0.787 |
| Black Caribbean |  | |  | 0.71 (0.44 - 1.13) | 0.157 | 0.71 (0.44 - 1.14) | 0.161 |
| Other Black |  | |  | 1.37 (0.69 – 2.74) | 0.363 | 1.36 (0.68 – 2.72) | 0.373 |
| Mixed/multiple |  | |  | 1.20 (0.69 - 2.09) | 0.515 | 1.19 (0.68 - 2.08) | 0.526 |
| Any other |  | |  | 0.93 (0.52 - 1.63) | 0.803 | 0.92 (0.52 - 1.62) | 0.778 |
| Black African |  | |  | 0.71 (0.48 - 1.04) | 0.084 | 0.70 (0.48- 1.04) | 0.082 |
| Non-British White |  | |  | 0.90 (0.53 - 1.53) | 0.702 | 0.89 (0.52 - 1.51) | 0.678 |
| Mixed White & Black |  | |  | 1.04 (0.63 – 1.72) | 0.87 | 1.04 (0.63 – 1.71) | 0.866 |
| Latin American |  | |  | 1.22 (0.64 - 2.30) | 0.537 | 1.21 (0.64 - 2.28) | 0.554 |
| **Receives free-school meals** |  | |  |  |  |  |  |
| No |  | |  | ***Ref*** |  | ***Ref*** |  |
| Yes |  | |  | 1.47 (1.09 – 1.97) | 0.010 | 1.46 (1.09 - 1.96) | 0.004 |
| **Social network control variable** |  | |  |  |  |  |  |
| Friendship group size |  | |  |  |  | 0.97 (0.91 - 1.04) | 0.455 |
|  | N = 2203 | | | N = 2203 | | N = 2203 | |

Model 1 is an unadjusted univariable model assessing the association between friend’s self-harm with lifetime self-harm. Model 2 is adjusted for gender, age, free school meal status, and ethnic group. Model 3 is further adjusted for friendship group size.

**Table S9.2 Estimated odds ratios for the effect of friendship group density and lifetime self-harm among adolescents overall**

|  | **Model 1** | | **Model 2** | | **Model 3** | |
| --- | --- | --- | --- | --- | --- | --- |
|  | **uOR (95%CI)** | **p** | **aOR (95%CI)** | **p** | **aOR (95%CI)** | **p** |
| **Friendship group density** |  |  |  |  |  |  |
| Medium density | ***Ref*** |  | ***Ref*** |  | ***Ref*** |  |
| Low density | 0.86 (0.66 - 1.12) | 0.281 | 0.94 (0.72 - 1.24) | 0.710 | 0.91 (0.70 - 1.23) | 0.639 |
| High density | 0.59 (0.36 - 0.97) | 0.039 | 0.50 (0.30 - 0.83) | 0.007 | 0.51 (0.31 - 0.85) | 0.010 |
| **Adolescent's characteristics** |  |  |  |  |  |  |
| **Gender** |  |  |  |  |  |  |
| Boy |  |  | ***Ref*** |  | ***Ref*** |  |
| Girl |  |  | 1.71 (1.29 - 2.28) | <.001 | 1.71 (1.28 - 2.27) | <.001 |
| **Age** |  |  | 1.25 (1.09 - 1.43) | 0.001 | 1.25 (1.09 - 1.43) | 0.001 |
| **Ethnicity** |  |  |  |  |  |  |
| White British |  |  | ***Ref*** |  | ***Ref*** |  |
| Indian, Pakistani, Bangladeshi |  |  | 0.86 (0.45 - 1.65) | 0.656 | 0.87 90.45 - 1.68) | 0.691 |
| Black Caribbean |  |  | 0.74 (0.45 - 1.19) | 0.218 | 0.73 (0.45 - 1.19) | 0.213 |
| Other Black |  |  | 1.24 (0.62 - 2.47) | 0.533 | 1.25 (0.63 - 2.49) | 0.518 |
| Mixed/multiple |  |  | 1.17 (0.67 - 2.04) | 0.564 | 1.18 (0.67 - 2.05) | 0.553 |
| Any other |  |  | 0.90 (0.51 - 1.60) | 0.737 | 0.91 (0.51 - 1.61) | 0.758 |
| Black African |  |  | 0.67 (0.44 - 1.00) | 0.055 | 0.67 (0.44 - 1.01) | 0.058 |
| Non-British White |  |  | 0.95 (0.56 - 1.61) | 0.861 | 0.95 (0.56 - 1.62) | 0.877 |
| Mixed White & Black |  |  | 1.02 (0.62 - 1.69) | 0.916 | 1.02 (0.62 - 1.69) | 0.913 |
| Latin American |  |  | 1.16 (0.60 - 2.21) | 0.650 | 1.16 (0.61 - 2.22) | 0.635 |
| **Receives free-school meals** |  |  |  |  |  |  |
| No |  |  | ***Ref*** |  | ***Ref*** |  |
| Yes |  |  | 1.57 (1.17 - 2.11) | 0.002 | 1.58 (1.17 - 2.12) | 0.002 |
| **Social network control variable** |  |  |  |  |  |  |
| Friendship group size |  |  |  |  | 1.01 (0.95 - 1.08) | 0.590 |
|  |  |  |  |  |  |  |
|  | N = 2203 | | N = 2203 | | N = 2203 | |

Model 1 is an unadjusted univariable model assessing the association between friendship group density with lifetime self-harm. Model 2 is adjusted for gender, age, free school meal status, and ethnic group. Model 3 is further adjusted for friendship group size.

**Table S9.3. Estimated odds ratios for the effect of bridging and lifetime self-harm among adolescents overall**

|  | **Model 1** | | **Model 2** | | **Model 3** | |
| --- | --- | --- | --- | --- | --- | --- |
|  | **uOR (95%CI)** | **p** | **aOR (95%CI)** | **p** | **aOR (95%CI)** | **p** |
| **Bridging** | 1.02 (1.01 - 1.04) | 0.008 | 1.02 (1.01 - 1.04) | 0.007 | 1.02 (1.01 - 1.04) | 0.014 |
| **Adolescent's characteristics** |  |  |  |  |  |  |
| **Gender** |  |  |  |  |  |  |
| Boy |  |  | ***Ref*** |  | ***Ref*** |  |
| Girl |  |  | 1.64 (1.23 - 2.17) | 0.001 | 1.64 (1.23 - 2.17) | 0.001 |
| **Age** |  |  | 1.24 (1.08 - 1.41) | 0.001 | 1.24 (1.08 - 1.41) | 0.001 |
| **Ethnicity** |  |  |  |  |  |  |
| White British |  |  | ***Ref*** |  | ***Ref*** |  |
| Indian, Pakistani, Bangladeshi |  |  | 0.89 (0.46 - 1.72) | 0.746 | 0.89 (0.46 - 1.72) | 0.747 |
| Black Caribbean |  |  | 0.76 (0.47 - 1.23) | 0.268 | 0.76 (0.47 - 1.23) | 0.268 |
| Other Black |  |  | 1.33 (0.66 - 2.67) | 0.410 | 1.33 (0.66 - 2.67) | 0.410 |
| Mixed/multiple |  |  | 1.16 (0.66 - 2.02) | 0.592 | 1.16 (0.66 - 2.02) | 0.592 |
| Any other |  |  | 0.93 (0.52 - 1.66) | 0.820 | 0.93 (0.52 - 1.66) | 0.820 |
| Black African |  |  | 0.69 (0.46 - 1.05) | 0.091 | 0.69 (0.46 - 1.05) | 0.091 |
| Non-British White |  |  | 0.97(0.57 - 1.66) | 0.934 | 0.97 (0.57 - 1.66) | 0.934 |
| Mixed White & Black |  |  | 1.04 (0.62 - 1.72) | 0.874 | 1.04 (0.62 - 1.72) | 0.874 |
| Latin American |  |  | 1.19 (0.62 - 2.28) | 0.594 | 1.19 (0.62 - 2.28) | 0.594 |
| **Receives free-school meals** |  |  |  |  |  |  |
| No |  |  | ***Ref*** |  | ***Ref*** |  |
| Yes |  |  | 1.57 (1.17 - 2.11) | 0.003 | 1.57 (1.17 - 2.11) | 0.003 |
| **Social network control variable** |  |  |  |  |  |  |
| Friendship group size |  |  |  |  | 1.00 (0.93 - 1.06) | 0.990 |
|  | N = 2203 | | N = 2203 | | N = 2203 | |

Model 1 is an unadjusted univariable model assessing the association between bridging with lifetime self-harm. Model 2 is adjusted for gender, age, free school meal status, and ethnic group. Model 3 is further adjusted for friendship group size.

**Table S9.4.** **Estimated odds ratios for the effect of popularity and lifetime self-harm among adolescents overall**

|  | **Model 1** | | **Model 2** | | **Model 3** | |
| --- | --- | --- | --- | --- | --- | --- |
|  | **uOR (95%CI)** | **p** | **aOR (95%CI)** | **p** | **aOR (95%CI)** | **p** |
| **Popularity** | 1.04 (0.99 - 1.10) | 0.082 | 1.04 (0.99 - 1.11) | 0.088 | 1.05 (0.99 - 1.12) | 0.051 |
| **Adolescent's characteristics** |  |  |  |  |  |  |
| **Gender** |  |  |  |  |  |  |
| Boy |  |  | ***Ref*** |  | ***Ref*** |  |
| Girl |  |  | 1.60 (1.21 - 2.12) | 0.001 | 1.62 (1.22 - 2.15) | 0.001 |
| **Age** |  |  | 1.23 (1.08 - 1.40) | 0.002 | 1.22 (1.07 - 1.39) | 0.003 |
| **Ethnicity** |  |  |  |  |  |  |
| White British |  |  | ***Ref*** |  | ***Ref*** |  |
| Indian, Pakistani, Bangladeshi |  |  | 0.89 (0.46 - 1.71) | 0.735 | 0.88 (0.46 – 1.70) | 0.717 |
| Black Caribbean |  |  | 0.75 (0.46 - 1.21) | 0.243 | 0.74 (0.46 - 1.20) | 0.227 |
| Other Black |  |  | 1.31 (0.65 – 2.62) | 0.435 | 1.29 (0.65 – 2.59) | 0.457 |
| Mixed/multiple |  |  | 1.15 (0.66 - 2.01) | 0.603 | 1.15 (0.66 - 2.00) | 0.616 |
| Any other |  |  | 0.90 (0.51 - 1.60) | 0.728 | 0.89 (0.50 - 1.58) | 0.708 |
| Black African |  |  | 0.67 (0.44 - 1.01) | 0.059 | 0.66 (0.44 – 0.99) | 0.049 |
| Non-British White |  |  | 0.95 (0.56 – 1.62) | 0.870 | 0.94 (0.55 - 1.60) | 0.844 |
| Mixed White & Black |  |  | 1.04 (0.63 – 1.72) | 0.861 | 1.04 (0.63 – 1.72) | 0.865 |
| Latin American |  |  | 1.21 (0.63 - 2.31) | 0.554 | 1.21 (0.63 - 2.53) | 0.559 |
| **Receives free-school meals** |  |  |  |  |  |  |
| No |  |  | ***Ref*** |  | ***Ref*** |  |
| Yes |  |  | 1.56 (1.16 - 2.10) | 0.003 | 1.57 (1.16 - 2.10) | 0.003 |
| **Social network control variable** |  |  |  |  |  |  |
| Sociality |  |  |  |  | 0.95 (0.88 – 1.02) | 0.192 |
|  | N = 2203 | | N = 2203 | | N = 2203 | |

Model 1 is an unadjusted univariable model assessing the association between popularity with lifetime self-harm. Model 2 is adjusted for gender, age, free school meal status, and ethnic group. Model 3 is further adjusted for sociality.

**Table S9.5. Estimated odds ratios for the effect of popularity and lifetime self-harm among adolescents overall**

|  | **Model 1** | | **Model 2** | | **Model 3** | |
| --- | --- | --- | --- | --- | --- | --- |
|  | **uOR (95%CI)** | **p** | **aOR (95%CI)** | **p** | **aOR (95%CI)** | **p** |
| **Sociality** | 0.97 (0.90 - 1.04) | 0.445 | 0.96 (0.89 - 1.04) | 0.376 | 0.95 (0.88 - 1.02) | 0.192 |
| **Adolescent's characteristics** |  |  |  |  |  |  |
| **Gender** |  |  |  |  |  |  |
| Boy |  |  | ***Ref*** |  | ***Ref*** |  |
| Girl |  |  | 1.65 (1.24 - 2.19) | <.001 | 1.62 (1.22 - 2.15) | 0.001 |
| **Age** |  |  | 1.22 (1.07 - 1.40) | 0.002 | 1.22 (1.07 - 1.39) | 0.003 |
| **Ethnicity** |  |  |  |  |  |  |
| White British |  |  | ***Ref*** |  | ***Ref*** |  |
| Indian, Pakistani, Bangladeshi |  |  | 0.84 (0.44 - 1.62) | 0.616 | 0.88 (0.46 – 1.70) | 0.717 |
| Black Caribbean |  |  | 0.75 (0.46 - 1.22) | 0.255 | 0.74 (0.46 - 1.20) | 0.227 |
| Other Black |  |  | 1.26 (0.63 - 2.51) | 0.505 | 1.29 (0.65 – 2.59) | 0.457 |
| Mixed/multiple |  |  | 1.14 (0.65 - 1.98) | 0.640 | 1.15 (0.66 - 2.00) | 0.616 |
| Any other |  |  | 0.87 (0.49 - 1.55) | 0.657 | 0.89 (0.50 - 1.58) | 0.708 |
| Black African |  |  | 0.66 (0.43 - 0.99) | 0.047 | 0.66 (0.44 – 0.99) | 0.049 |
| Non-British White |  |  | 0.93 (0.55 - 1.58) | 0.804 | 0.94 (0.55 - 1.60) | 0.844 |
| Mixed White & Black |  |  | 1.04 (0.63 - 2.23) | 0.626 | 1.04 (0.63 – 1.72) | 0.865 |
| Latin American |  |  | 1.17 (0.61 - 2.23) | 0.626 | 1.21 (0.63 - 2.53) | 0.559 |
| **Receives free-school meals** |  |  |  |  |  |  |
| No |  |  | ***Ref*** |  | ***Ref*** |  |
| Yes |  |  | 1.54 (1.15 - 2.07) | 0.004 | 1.57 (1.16 - 2.10) | 0.003 |
| **Social network control variable** |  |  |  |  |  |  |
| In-degree |  |  |  |  | 1.05 (0.99 - 1.12) | 0.051 |
|  | N = 2203 | | N = 2203 | | N = 2203 | |

Model 1 is an unadjusted univariable model assessing the association between sociality with lifetime self-harm. Model 2 is adjusted for gender, age, free school meal status, and ethnic group. Model 3 is further adjusted for popularity.

**Table S9.6. Estimated odds ratios for the effect of isolation and lifetime self-harm among adolescents overall**

|  | **Model 1** | | **Model 2** | |
| --- | --- | --- | --- | --- |
|  | **uOR (95%CI)** | **p** | **aOR (95%CI)** | **p** |
| **Isolation** |  |  |  |  |
| No | ***Ref*** |  | ***Ref*** |  |
| Yes | 1.73 (1.05 - 2.83) | 0.029 | 1.86 (1.12 - 3.08) | 0.016 |
| **Adolescent's characteristics** |  |  |  |  |
| **Gender** |  |  |  |  |
| Boy |  |  | ***Ref*** |  |
| Girl |  |  | 1.68 (1.27 - 2.23) | <.001 |
| **Age** |  |  | 1.22 (1.07 - 1.39) | 0.003 |
| **Ethnicity** |  |  |  |  |
| White British |  |  | ***Ref*** |  |
| Indian, Pakistani, Bangladeshi |  |  | 0.83 (0.43 - 1.60) | 0.587 |
| Black Caribbean |  |  | 0.75 (0.46 - 1.22) | 0.743 |
| Other Black |  |  | 1.21 (0.60 - 2.41) | 0.584 |
| Mixed/multiple |  |  | 1.12 (0.64 - 1.96) | 0.67 |
| Any other |  |  | 0.86 (0.48 - 1.53) | 0.622 |
| Black African |  |  | 0.65 (0.43 - 0.97) | 0.039 |
| Non-British White |  |  | 0.93 (0.54 - 1.57) | 0.790 |
| Mixed White & Black |  |  | 1.04 (0.63 - 1.72) | 0.863 |
| Latin American |  |  | 1.19 (0.62 - 2.27) | 0.591 |
| **Receives free-school meals** |  |  |  |  |
| No |  |  | ***Ref*** |  |
| Yes |  |  | 1.56 (1.16 - 2.09) | 0.003 |
|  | N = 2203 | | N = 2203 | |

Model 1 is an unadjusted univariable model assessing the association between isolation with lifetime self-harm. 
Model 2 is adjusted for gender, age, free school meal status, and ethnic group.

**S10. Additional notes from YPCC discussion**

Five young people from the YPCC helped to brainstorm about the potential interpretation of findings of this work. Among the YPCC, there was a real sense that this work addressed an important research question, the findings were novel, and that peer-friendships are crucial to consider for self-harm in adolescence. Importantly, the young people reflected that the visualisations of the networks (sociograms) were visually engaging and gave real insight into the lives of school-based adolescents in REACH. Young people reflected on how the sociograms made them think about their own experiences of being at school. They suggested the sociograms helped them to understand more intuitively what the structure of school year groups might look like, and how adolescents might hold different positions within their school year group. Thus, the young people saw real value in the description and visualisation of the friendship networks. This further reinforces the benefits of mapping out networks visually as an important first step when analysing new sociometric data.

The YPCC had interesting reflections concerning the association between betweenness centrality (i.e., bridging others) and increased odds of lifetime self-harm among adolescents. Young people suggested that adolescents in extreme bridging positions are not necessarily “here nor there” and that being between others might be a compromising position to hold and taxing on mental wellbeing. One young person suggested this finding could be because bridging others in a school year group might mean that an adolescent are “isolated” from the two groups that they are bridging (i.e., not quite embedded in either friendship group, but still embedded within the school year group).
